# Late-Onset Cognitive Impairments after Early-Life Stress Are Shaped by Inherited Differences in Stress Reactivity

**DOI:** 10.3389/fncel.2017.00009

**Published:** 2017-02-14

**Authors:** Silja McIlwrick, Tobias Pohl, Alon Chen, Chadi Touma

**Affiliations:** ^1^Department of Stress Neurobiology and Neurogenetics, Max Planck Institute of PsychiatryMunich, Germany; ^2^Department of Neurobiology, Weizmann Institute of ScienceRehovot, Israel; ^3^Department of Behavioural Biology, University of OsnabrückOsnabrück, Germany

**Keywords:** stress reactivity mouse model, early-life stress, gene × environment interaction, HPA axis, cognition, BDNF

## Abstract

Early-life stress (ELS) has been associated with lasting cognitive impairments and with an increased risk for affective disorders. A dysregulation of the hypothalamus-pituitary-adrenal (HPA) axis, the body’s main stress response system, is critically involved in mediating these long-term consequences of adverse early-life experience. It remains unclear to what extent an inherited predisposition for HPA axis sensitivity or resilience influences the relationship between ELS and cognitive impairments, and which neuroendocrine and molecular mechanisms may be involved. To investigate this, we exposed animals of the stress reactivity mouse model, consisting of three independent lines selectively bred for high (HR), intermediate (IR), or low (LR) HPA axis reactivity to a stressor, to ELS and assessed their cognitive performance, neuroendocrine function and hippocampal gene expression in early and in late adulthood. Our results show that HR animals that were exposed to ELS exhibited an HPA axis hyper-reactivity in early and late adulthood, associated with cognitive impairments in hippocampus-dependent tasks, as well as molecular changes in transcript levels involved in the regulation of HPA axis activity (*Crh*) and in neurotrophic action (*Bdnf*). In contrast, LR animals showed intact cognitive function across adulthood, with no change in stress reactivity. Intriguingly, LR animals that were exposed to ELS even showed significant signs of enhanced cognitive performance in late adulthood, which may be related to late-onset changes observed in the expression of *Crh* and *Crhr1* in the dorsal hippocampus of these animals. Collectively, our findings demonstrate that the lasting consequences of ELS at the level of cognition differ as a function of inherited predispositions and suggest that an innate tendency for low stress reactivity may be protective against late-onset cognitive impairments after ELS.

## Introduction

Many affective disorders have their roots in the perinatal phase of development, when important networks in the central nervous system (CNS) are being shaped (Heim and Nemeroff, 2002; Provencal and Binder, 2015). During this period, the CNS is particularly sensitive to environmental cues, so that key signaling pathways and neuronal ensembles in the brain can be lastingly programmed in their response to relevant stimuli (Bale et al., 2010; Barker et al., 2013). This offers an important window of opportunity to prepare a developing organism for challenges in its future environment. Epigenetic processes, that can modify gene expression for example through DNA methylation or histone modification without changing the genetic code, are thought to play a central role in this process (Meaney et al., 2007; Murgatroyd and Spengler, 2011; Heim and Binder, 2012). Early-life programming may be adaptive in some cases [e.g., the attenuation of the enzyme 11β-HSD 2 can be beneficial to regulate sodium retention in nutrient poor environments (Yehuda and Seckl, 2011)]. However, studies in both humans and animal models have shown that programming in response to early-life stress (ELS) exposure can precipitate a dysregulation of the hypothalamic-pituitary-adrenal (HPA) axis in later life (Heim et al., 2000; Shea et al., 2005; Korosi and Baram, 2010; McIlwrick et al., 2016). This can be evidenced by alterations in the corticotropin-releasing hormone (CRH) system, exaggerated release of glucocorticoid hormones from the adrenal cortex in response to stressors, and by impaired negative feedback via glucocorticoid and mineralocorticoid receptors (GR and MR) in the brain. Dysregulation of the HPA axis can have detrimental consequences for future health and coping (Meaney, 2001; Pryce and Feldon, 2003), as it significantly increases the risk for affective disorders, including major depressive disorders (MDD) and anxiety disorders (Holsboer, 1999, 2000; Sanchez et al., 2001; Yehuda and Seckl, 2011; Heim and Binder, 2012).

Limbic brain areas, such as the hippocampal formation, are particularly sensitive to the signaling of stress hormones, as they abundantly express GRs and MRs, as well as CRH receptor type 1 (CRH-R1) (de Kloet et al., 1998; Joels and Baram, 2009; Henckens et al., 2016). High levels of glucocorticoids can activate signaling pathways that exert neurotoxic effects when excessively or chronically activated (Raber, 1998; Conrad, 2008). Importantly, the hippocampus develops perinatally, so that ELS can directly impact on its development. Many studies have shown that the excitotoxic effects of stress, or of exposure to excessive levels of glucocorticoids, include reduced survival of newborn cells (Sapolsky, 1985; Gould et al., 1991; Naninck et al., 2015), downregulation of brain derived neurotrophic factor (BDNF) (Daskalakis et al., 2015) and reduced synaptic plasticity (Anacker et al., 2011; Liston and Gan, 2011), as well as changes of dendritic morphology and dendritic atrophy of pyramidal neurons (Magarinos et al., 1996; McKittrick et al., 2000; Brunson et al., 2005; Alfarez et al., 2009). Imaging studies in MDD patients have extended these findings by demonstrating that a reduced hippocampal volume is associated with ELS experience (Kronmuller et al., 2008; Woon and Hedges, 2008; Teicher et al., 2012). In line with this, research has revealed reduced levels of BDNF in the hippocampus of ELS exposed mice and ELS has been causally linked to impaired hippocampus-dependent memory (Brunson et al., 2005; Nelson et al., 2007; Gould et al., 2012), which is suggested to occur after excessive stress-induced activation of hippocampal GR and subsequent changes in the CRH/CRHR1 system (Ivy et al., 2010; Wang et al., 2013). Together, the accumulated evidence demonstrates that ELS can alter HPA axis function, thereby adversely affecting the integrity and function of the hippocampus, and ultimately increasing the risk for affective disorders. However, genetic factors also play an important role in the etiology affective disorders (Kendler, 2001; Lohoff, 2010) and are likely moderators of individual trajectories toward resilience or vulnerability. Evidence suggests that individuals with an inherited predisposition for a dysregulation of the stress hormone system may be at greater risk for major MDD (Holsboer, 1999; Pariante and Lightman, 2008). Interestingly, within the group of patients diagnosed with MDD, two distinct subtypes can be distinguished based on the profile of their HPA axis function (Gold and Chrousos, 1999, 2002; Antonijevic, 2006). On one extreme are patients with HPA axis hyper-reactivity (psychotic or melancholic depression subtype), displaying symptoms such as restlessness, hyperactivity, a shift in their diurnal endocrine rhythms, impaired sleep architecture with increased REM sleep, weight loss, and cognitive impairments. On the other extreme are patients with a markedly reduced HPA axis reactivity, i.e., stress hypo-reactivity (atypical depression subtype), showing symptoms of lethargy, hypersomnia, weight gain and a heightened sensitivity for social rejection, but no signs of cognitive impairments. This stratification of MDD patients by HPA axis function suggests that different genetic predispositions may be underlying the divergent endophenotypes (Antonijevic, 2006; Heinzmann et al., 2014). The stress reactivity (SR) mouse model is a genetic animal model, which recapitulates several of the key endophenotypes of the two MDD subtypes described above (Touma et al., 2008; Heinzmann et al., 2014), including associated changes in bodyweight (Touma et al., 2009), sleep architecture (Touma et al., 2009; Fenzl et al., 2011), stress hormone profiles (Touma et al., 2008, 2009; Heinzmann et al., 2014), and cognitive performance (Knapman et al., 2010a,b, 2012). Using this animal model, our group recently showed that an inherited predisposition for extremes in stress reactivity (high or low) interacts with ELS to shape short-term, as well as lasting consequences at the level of stress-coping behavior, neuroendocrine function, and gene expression (McIlwrick et al., 2016). It remained uncertain, however, to what extent this gene x environment interaction has long-term effects on cognitive function and on the expression of important neurotrophic factors in the hippocampus. To investigate this question, we exposed animals of the three SR mouse lines to a well-established paradigm of ELS, based on limited nesting and bedding material (Rice et al., 2008), and assessed their cognitive performance using several tests during early and late adulthood, as evidence points toward cumulative effects of glucocorticoid exposure over time. In addition, we measured the relative expression of selected candidate genes that have been implicated in the effects of ELS on cognitive function [*Bdnf, Ntrk2* (TrkB), *Crh, Crh-r1*] in the dorsal and ventral hippocampus at both time points. The animals’ bodyweight and their HPA axis reactivity were also assessed to validate the model and to monitor the long-lasting programming effects of the ELS paradigm.

## Materials and Methods

All presented work is in accordance with the accepted ethical standards of humane care and use of experimental animals and was approved by the appropriate local authority (Regierung Oberbayern, Verbraucherschutz und Veterinärwesen, Sachgebiet 54, project code 55.2-1-54-2532-148-2012).

### The Stress Reactivity Mouse Model

The SR mouse model consists of three independent mouse lines selectively bred for either high (HR) or low (LR) HPA axis reactivity in response to a psychological stressor. The IR mouse line, bred for intermediate stress reactivity, serves as a reference line. Briefly, to generate this animal model, a founder generation of 100 outbred CD-1 male and female mice was tested in the stress reactivity test (SRT), which measures the animal’s CORT release in response to a psychological stressor (15 min restraint, a detailed description of the SRT is provided in section “Stress Reactivity and CORT Measurement”). Based on the test results, breeding pairs were selected to generate the HR, IR, and LR mouse lines. Through repeated testing and re-selection of every new generation at the age of 7–8 weeks, three inbred mouse lines were established. The SR mouse model has been extensively phenotyped and several parallels regarding symptoms associated with MDD have been highlighted. In the HR line, these include a reduced bodyweight, increased locomotor activity, hyperactive stress-coping behavior, altered sleep architecture with increased rapid eye movement (REM) sleep, and impaired cognition, akin to endophenotypes of melancholic/psychotic depression. On the other hand, LR animals show an increased bodyweight, reduced locomotor activity, passive stress-coping behavior, and intact sleep and cognitive function, in line with endophenotypes of the atypical depression subtype (Touma et al., 2008, 2009; Knapman et al., 2010a,b, 2012; Fenzl et al., 2011; Pillai et al., 2012; Heinzmann et al., 2014).

### The Early-Life Stress Paradigm

To induce ELS, we used the limited nesting and bedding material paradigm (Rice et al., 2008), which creates a stressful early-life situation for the dam and her pups, without having to physically remove the dam from the litter. This ELS paradigm has been described as more ecologically valid than maternal separation (Molet et al., 2014) and its lasting effect on the offspring has been replicated in rats and mice in several studies (Avishai-Eliner et al., 2008; Gunn et al., 2013; Machado et al., 2013; Naninck et al., 2015; McIlwrick et al., 2016). Briefly, 16 dams of each SR mouse line were randomly assigned to either the ELS or the standard (STD) housing condition. On P2, after regulating the litters (see Breeding of Experimental Animals), dams in the ELS condition were placed, together with their pups, into a macrolone cage type II, the floor of which was covered by an aluminum grid (mesh dimensions 0.4 cm × 0.9 cm, catalog no. 57398; McNichols Co., Tampa, FL, USA). Twenty grams of wood chip bedding and half a nestlet (∼5 g) were provided for nest building. Dams in the STD condition were moved, together with their pups, into standard macrolone cages type II with ample wood chip bedding (∼100 g) and 2 nestlets (∼20 g) for nest building. The animals were then left undisturbed until P9, when all litters were moved to standard cages, where they stayed with their mother until weaning (P25). In a previous study using this ELS paradigm in the SR mouse model, we conducted a detailed analysis of the maternal behavior (McIlwrick et al., 2016). Briefly, the dams of all three mouse lines show similar changes in their maternal behavior (number of exits and time spent on the nest) when rearing their pups in the ELS condition. Emerging differences between the pups can thus not be attributed to differences in maternal care between the three mouse lines.

### Experimental Design

To investigate the gene x environment interaction of a genetic predisposition for extremes in stress reactivity with ELS, we used a three-by-two experimental design (i.e., the three SR mouse lines, HR, IR and LR, and two conditions, ELS and STD), thus resulting in a total of six experimental groups. This two-factorial design was employed both when testing animals during early and late adulthood. In total, the data presented in this manuscript was collected from three sequential cohorts of experimental animals, generated from breeding generations XXIII, XXVI, and XXVII of the SR mouse model. Animals from experimental cohorts I and II were tested starting at 16 ± 1 weeks of age (early adulthood), while animals from cohort III were tested starting at 26 ± 1 weeks of age (late adulthood). There was some natural stratification in the birth dates of the litters of each breeding cohort (∼14 days), so that the mean age of the animals in each cohort was used to determine the date to start testing. An overview and timeline of the experiments included in this study, and of the number of animals used, is provided in **Supplementary Figure S1**.

### Animals

For this study, male animals from the three SR mouse lines (HR, IR, and LR) were used. All mice were bred in-house, and housed in sibling-pairs until 2 weeks before the behavioral testing started, when they were single housed to avoid influences of dominance hierarchy. Mice were housed in macrolone cages under standard laboratory conditions, with standard chow and water *ad libitum*, a 12 h light/dark cycle (lights on at 8:00) and constant humidity (55 ± 10%). Cages were changed once per week, but never on the day of or immediately before testing.

#### Breeding of Experimental Animals

For breeding of each cohort of animals, 16–20 females and 8–10 males of each of the three SR mouse lines (HR, IR, and LR) were housed in triplets (two females, one male) for 14 days to allow mating. Thereafter, the females were moved to fresh single cages with ample nesting material. Cages were checked every day at 17:00 for the delivery of litters. The day a litter was discovered was defined as postnatal day 0 (P0). On P2, litters were culled to seven pups (including at least five males), to maximize the similarity in the early-life situation between litters. Litters with less than five pups and litters with only same-sex pups were not included in the experiment. On P25, pups were weaned and pair-housed with same-sex siblings until adulthood.

### Bodyweight

During and after the ELS paradigm, the animals’ bodyweight was closely monitored. Research has shown that bodyweight can be lastingly affected by stress manipulations during early-life (reviewed in Maniam et al., 2014), making this readout a valuable indicator of the impact of the ELS paradigm. The mean pup weight per litter was assessed on P2, P9, P17, and P25, and in adulthood bodyweight was measured 2 weeks before the behavioral testing.

### Behavioral and Cognitive Testing

All tests were performed between 08:00 and 13:00 h, when the animals’ CORT levels are in the circadian trough (Ishida et al., 2005; Touma et al., 2009). Between the different behavioral tests, the animals were allowed at least 48 h of rest in order to avoid carry-over effects influencing behavioral readouts in subsequent tests (McIlwain et al., 2001). During the tests, the animals’ behavior was video-recorded and tracked using automated tracking software (ANY-maze, Stoelting GmbH), with the exception of the water cross-maze test, which was scored in real-time by a trained observer. At the beginning and between different animals, the testing apparatus was cleaned with soapy water and 10% ethanol solution and dried to remove any odor cues.

#### Open Field Test

We used the open field test (OFT) to assess the animals’ exploratory and anxiety-related behavior. In this classic behavioral test (for review see Bailey and Crawley, 2009) the animal is placed into the center of a circular arena (∅ 60 cm), which is dimly lit (15 Lux), and is allowed to explore freely for 5 min. Behavioral measures of exploration include the total distance traveled by the animal in the arena, as well as the time and distance it traveled in the more aversive central zone (∅ 30 cm). For animal cohort I, tested during early adulthood, the OFT was the first in a battery of behavioral tests. In cohort III, tested during late adulthood, the OFT was incorporated into the habituation phase for the object recognition test (see Object Recognition Test). The entire habituation phase in the OF arena lasted for 20 min. To compare the results with those from the early adulthood cohort, we extracted the data from the first 5 min and analyzed these separately.

#### Water Cross-Maze Test

The water cross-maze (WCM) is a test for hippocampus-dependent learning and memory. It was described in detail by Kleinknecht et al. (2012). Briefly, the apparatus consists of a cross-shaped maze, made of transparent acrylic glass (maze dimensions: arm length: 50 cm, arm width: 10 cm, wall height: 30 cm), which is filled with water (∼23°C, 11 cm deep). The maze was placed on a wooden platform (30 cm above the floor) in an evenly and dimly lit room (∼14 Lux), containing some environmental spatial cues (shelves, ceiling pipes). We used a place learning protocol as described previously (Kleinknecht et al., 2012) to assess the animals’ spatial memory performance. Briefly, the cross-maze was converted to a T-maze by blocking the arm opposite to the start arm with an acrylic glass shield. Each animal was gently placed into the water facing the back wall of the start arm and had to swim to a location where a small platform (acrylic glass, 8 cm × 8 cm) was submerged under the water surface at the end of the goal arm. Once the mouse had climbed onto the hidden platform, it was removed from the water, dried and returned to its home cage (which was placed partly under an infrared lamp for voluntary heating). If an animal failed to locate the platform within 30 s, it was manually guided to the platform and 31 s was entered as latency for this trial. In all other cases, the experimenter remained motionless behind the start arm until the animal had reached the platform, so as not to provide any cue for the platform location. Each animal completed six trials per day on eight consecutive days, with an inter-trial interval (ITI) of ∼10 min. As required by the place learning protocol, the start arm varied in a pseudo-random order, while the hidden platform always remained in the same position. Thus, the animals had to make use of distal spatial cues in order to minimize the time to reach their target. This place learning strategy involves building a cognitive map of the environment and is dependent on intact hippocampus function (O’Keefe et al., 1975; Morris et al., 1982; Gutierrez-Guzman et al., 2011). To assess the animals’ performance, three main variables were quantified in each trial: (1) accuracy (scored as 0 if the animals entered any other arm before entering the goal arm, scored as 1 if the animal directly entered the goal arm from the start arm), (2) latency (the time from entering the water to climbing onto the hidden platform), (3) number of wrong platform visits [scored as 0 if the animal did not enter the outer third of any arm apart from the goal arm, scored as 1 (or more) if the animal swam into the outer third of any non-goal arm]. For the analysis, the scores on all six daily trials were averaged per animal on every training day. The WCM test was used to assess spatial learning only in animals during early adulthood (cohort I).

#### Y-Maze Test

The Y-maze is a frequently used behavioral test to assess hippocampus-dependent spatial memory in rodents (Dellu et al., 2000). The test is based on the innate tendency of rodents to explore unfamiliar areas. The apparatus consists of a Y-shaped maze (three arms joining in a central area, arm length: 30 cm, arm width: 11 cm, wall height: 15 cm) made of dark gray plastic, evenly illuminated with 15 Lux. The walls of each of the three arms were marked with a white symbol (a triangle, a bar or a plus), so that they could be clearly distinguished. The test consisted of an acquisition phase (10 min), followed by an ITI of 60 min, and a retrieval phase (5 min). During the acquisition phase, the plus-arm was blocked by a removable wall. The animal was placed into the central area, facing the corner joining the two open arms together, and was allowed to freely explore the maze. In the ITI, the animal was returned to its home cage. For the retrieval phase, the wall blocking the plus-arm was removed; the mouse was again placed into the center area and allowed to explore the entire maze. To derive a measure of cognitive performance, a discrimination ratio was calculated using the following formula: (distance in the novel arm – the mean exploration distance in the two familiar arms)/total distance in all three arms. The discrimination ratio provides a measure of whether an animal distinguished between the novel and the familiar arms (i.e., if the ratio is larger than zero). It is also possible to calculate a discrimination ratio based on the time the animal spent in each of the arms (we provide both measures here), but this time-based discrimination ratio may sometimes be less sensitive to detect subtle differences in task performance, as animals can spend a lot time sitting in one arm, without actually exploring it. We used the Y-maze test to assess spatial memory in mice during both early and late adulthood (cohorts II and III).

#### Object Recognition Test

The ORT is one of the most frequently used tests for non-spatial memory in rodents (Akkerman et al., 2012). The performance in the ORT is dependent on hippocampal function (Clark et al., 2000), as well as on perirhinal and entorhinal cortex activity (Buckmaster et al., 2004; Aggleton et al., 2010). Similar to the Y-maze test, the ORT relies on the animals’ natural preference for novelty. We followed the testing protocol described by Leger et al. (2013). Briefly, 24 h before the familiarization phase, each animal was placed into the open field arena to freely explore for 20 min to allow habituation and to reduce the stressfulness of the subsequent testing phases. On the next day, the animal was returned to the arena, where two identical objects (constructed from LEGO blocks, Lego Group, Billund, Denmark) had been placed and was allowed to explore for 10 min (familiarization phase). During the ITI (60 min) the animal was returned to its home cage. In the test phase, one familiar and one novel object (also built from LEGO blocks, but with a different shape and color) were placed at the identical locations to where the objects in the familiarization phase had been, and the animal was again allowed to explore for 10 min. The objects we used were same as those previously employed by Knapman et al. (2010a) and have been pretested to make sure they were equally “interesting” to the animals. To assess the animals’ memory performance, a discrimination ratio (Akkerman et al., 2012) was calculated [formula: (time exploring novel object – time exploring familiar object)/time exploring both objects]. Exploration was defined as the animals head being within a 3 cm circumference from the object’s center. Any animal that failed to explore any of the objects for less than 5 s during the familiarization was excluded from the analysis. The ORT was performed in animals during late adulthood (cohort III).

### Stress Reactivity and CORT Measurement

As a measure of HPA axis responsiveness we used the SRT, as previously described (Touma et al., 2008). Briefly, each mouse was removed from its home cage and an initial blood sample was obtained through a small incision from the ventral tail vessel (to ensure the reference sample was very close to baseline levels, the time between initial handling of the cage until completing the blood sampling was less than 2 min). The mouse was then placed into a small restrainer (50 ml plastic tube, with holes for ventilation and an aperture in the cap for the tail) for 15 min, whereafter it was decapitated (after a very brief isoflurane anesthesia), and a “reaction sample” was collected from the trunk blood. We employed the SRT to measure the animals’ stress reactivity during both early and late adulthood (cohorts II and III). All blood samples were kept on ice until centrifuged (4°C) and plasma was removed for measurement of CORT using radioimmunoassay, according to the manufacturer’s protocol (DRG Instruments GmbH, Marburg, Germany), with slight modifications (for details see Touma et al., 2008). All samples were measured in duplicates and the intra- and inter-assay coefficients of variation were both below 10%.

### Gene Expression

To detect lasting consequences of ELS exposure at the level of gene regulation in the three SR mouse lines, we analyzed the relative expression of selected candidate genes using quantitative polymerase chain reaction (qPCR). Early and late adulthood samples were collected from cohorts I and III to be able to see if the effects of ELS changed over time. Briefly, after the SRT on the last day of testing, the animals were decapitated, the brain was removed, snap frozen in iced methylbutane, and stored at -80°C until further processing. The brains were sectioned into 200 μm thick coronal slices and mounted onto glass slides. Tissue punches of the dorsal (-1.2 to -2,0 mm from Bregma) and ventral (-3.0 to -3,80 mm from Bregma) hippocampus (dHip and vHip) were collected via micropuncture (for further details see Heinzmann et al., 2014). Total mRNA was extracted using RNeasy columns (RNeasy Micro Kit, Qiagen, Hilden, Germany) and 200 ng of the extracted mRNA was reverse transcribed to cDNA using high-capacity transcription kits (High-Capacity cDNA Reverse Transcription Kit, Applied Biosystems, Foster City, CA, USA). The expression of candidate genes was measured using qPCR kits (QuantiFast SYBR Green, Qiagen GmbH, Hilden, Germany) following the manufacturer’s protocol. All samples were measured in duplicates [Standard deviation (SD) < 1.0] on 384 well-plates with three genes per plate, including standard curves. A list of all measured candidate genes with the applied oligonucleotide primers is provided in **Table 1**. The relative fold expression of each gene was calculated using the ΔΔCT method (Livak and Schmittgen, 2001) by normalizing to two housekeeping genes [TATA-binding protein (*Tbp*) and Hypoxanthine-Guanine Phosphoribosyltransferase (*Hprt*)] and again normalizing to the mean of IR STD group.

**Table 1:** List of candidate genes.

Candidate gene	Designation	Direction	Sequence	*T*_m_	Amplicon length (bp)
*Bdnf*	Brain-derived neurotrophic factor	Forward	GTGTGACAGTATTAGCGAGTG	57.4	144
		Reverse	GGATTACACTTGGTCTCGTAG	57.7	
*Ntrk2*	Tyrosine receptor kinase B	Forward	TTCTGGAGTTTCTGCCCCTG	59.6	294
		Reverse	GGACTCTTTGGGTCGCAGAA	60.0	
*Crh*	Corticotropin releasing hormone	Forward	GCATCCTGAGAGAAGTCCCTCTG	67.5	135
		Reverse	GCAGGACGACAGAGCCA	64.2	
*Crh-r1*	Corticotropin releasing hormone receptor 1	Forward	GGTCCTGCTGATCAACTTTA	59.2	152
		Reverse	ACATGTAGGTGATGCCCA	59.9	
*Hprt*	Hypoxanthine guanine phosphoribosyl transferase	Forward	GTTGGATACAGGCCAGACTTTGT	65.1	225
		Reverse	CCACAGGACTAGAACACCTGCTA	64.3	
*Tbp*	TATA box binding protein	Forward	CCCCCTTGTACCCTTCACC	65.4	285
		Reverse	TGGATTGTTCTTCACTCTTGG	65.3	

*Candidate genes measured by quantitative real-time polymerase chain reaction (qPCR), including full designation, oligonucleotide primer sequence, melting temperature (T_m_) and the amplicon length in base pairs (bp).*

### Statistical Analysis

All statistical data analysis was conducted in PASW 18 or in Python. We used a two-way analysis of variance (ANOVA) with the independent variables “line” and “condition” to assess the main effects and the interaction of these factors. To detect the difference between early and late adulthood data, “age” was added as an independent variable. When data points were collected repeatedly from the same animal in one test, a three-way repeated-measures ANOVA, with “line” and “condition” as between-subjects factors, and “sampling time” as a within-subjects variable, was employed. Where appropriate, *post hoc* tests were conducted and corrected using the Bonferroni method. The association between cognitive performance and gene expression was investigated using the Pearson’s correlation coefficient. Statistical significance was accepted for ^∗^*p* ≤ 0.05, ^∗∗^*p* ≤ 0.01, ^∗∗∗^*p* ≤ 0.001, while *p* ≤ 0.1 (T) was considered a trend.

Some data from cohort I animals was presented in a previous publication (McIlwrick et al., 2016) (OFT, expression of hippocampal *Crh* and *Crhr1*) and is shown here again, as we aimed to provide both an early and a late adulthood assessment for all read-outs, without increasing the number of animals sacrificed for this research.

## Results

### ELS Influences Bodyweight Development

**FIGURE 1:**
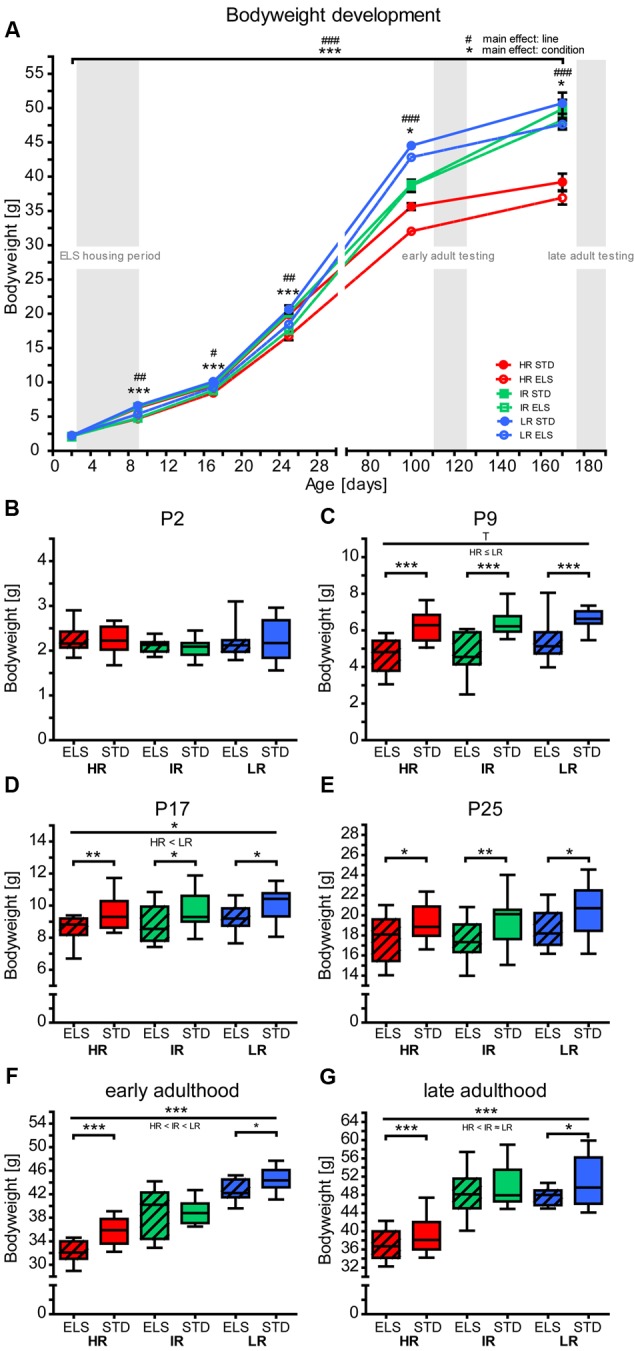
**Bodyweight development.** Bodyweight development of high (HR), intermediate (IR), and low (LR) reactivity mice, raised in early-life stress (ELS) or standard (STD) housing conditions was analyzed by repeated-measures or univariate ANOVA. Data are presented as line plots showing means and standard error of the mean (SEM) (error bars) and as boxplots showing the median (horizontal line in the box), 25–75% (boxes) and 10–90% (whiskers). Before weaning (i.e., on P2, P9, P17, and P25), pup weight is presented as the mean pup weight per litter. **(A)** The animals’ bodyweight shows different developmental trajectories. A repeated-measures ANOVA over all time points revealed a main effect of line (*F*_2,35_ = 87.404, *p* < 0.001, *post hoc* tests: P2: *p* = 0.208, P9: *p* = 0.002, P17: *p* = 0.020, P25: *p* = 0.005, P100: *p* < 0.001, P170: *p* < 0.001) and a main effect of condition (*F*_1,35_ = 45.631, *p* < 0.001, *post hoc* tests: P2: *p* = 0.872, P9: *p* < 0.001, P17: *p* < 0.001, P25: *p* < 0.001, P100: *p* = 0.031, P170: *p* = 0.018). **(B–G)** Show each time point in more detail: **(B)** On P2, there were no significant differences in bodyweight between the lines (*F*_2,93_ = 2.367, *p* = 0.099, *post hoc* tests: all *p* > 0.1) or conditions (*F*_1,93_ = 0.63, *p* = 0.803), *N*_(litters)_ = 14–18. **(C)** On P9, there was a statistical trend main effect of line on bodyweight (*F*_2,90_ = 2.846, *p* = 0.063, *post hoc* tests: HR vs. IR: *p* = 1.0, HR vs. LR: *p* = 0.086, IR vs. LR: *p* = 0.279), and a main effect of condition *F*_1,90_ = 71.557, *p* < 0.001, *post hoc* tests: all *p* < 0.001), *N*_(litters)_ = 14–17. **(D)** On P17, there was a main effect of line on bodyweight (*F*_2,87_ = 3.339, *p* = 0.040, *post hoc* tests: HR vs. IR: *p* = 1.0, IR vs. LR: *p* = 0.295, HR vs. LR: *p* = 0.036), and a main effect of condition (*F*_1,87_ = 20.396, *p* < 0.001, *post hoc* tests: HR ELS vs. STD: *p* = 0.004, IR ELS vs. STD: *p* = 0.012, LR ELS vs. STD: *p* = 0.023), *N*_(litters)_ = 14–16. **(E)** On P25, the main effect of line was not significant (*F*_2,87_ = 2.336, *p* = 0.103), but there was a main effect of condition (*F*_1,87_ = 18.181, *p* < 0.001, *post hoc* tests: HR ELS vs. STD: *p* = 0.032, IR ELS vs. STD: *p* = 0.007, LR ELS vs. STD: *p* = 0.016), *N*_(litters)_ = 14–17. **(F)** During early adulthood, there was a main effect of line (*F*_2,127_ = 169.490, *p* < 0.001, *post hoc* tests: all: *p* < 0.001), a main effect of condition (*F*_1,127_ = 17.543, *p* < 0.001), and an interaction of line and condition (*F*_2,127_ = 5.317, *p* = 0.007, *post hoc* tests: HR ELS vs. STD: *p* < 0.001, IR ELS vs. STD: *p* = 0.811, LR ELS vs. STD: *p* = 0.026), *N* = 22-23. **(G)** During late adulthood, there was a main effect of line (*F*_2,65_ = 54.141, *p* < 0.001, *post hoc* tests: HR vs. IR and HR vs. LR: *p* < 0.001, IR vs. LR: *p* = 1.0) and a main effect of condition (*F*_1,65_ = 5.331, *p* = 0.024, *post hoc* tests: HR ELS vs. STD: *p* = 0.181, IR ELS vs. STD: *p* = 0.349, LR ELS vs. STD: *p* = 0.096), *N* = 10–13. ^∗∗∗^ (or ###) *p* ≤ 0.001, ^∗∗^ (or ##) *p* ≤ 0.01, ^∗^ (or #) *p* ≤ 0.05, T, *p* ≤ 0.1. Main effects of line are represented above a horizontal line above the graphs. The respective *post hoc* test statistics are indicated underneath the line with: </>, *p* ≤ 0.05; ≤/≥, *p* ≤ 0.1; ≈, *p* > 0.1. *Post hoc* statistics for main effects of condition and the interaction are presented above the appropriate boxes.

**Figure 1A** illustrates the development of the animals’ bodyweight throughout the entire experimental time span. Before the start of the ELS paradigm, on P2, there were no significant differences in bodyweight between pups of the three mouse lines, or between pups assigned to the ELS or STD condition (**Figure 1B**). After 1 week of ELS or STD housing, on P9, the analysis revealed a significant main effect of condition (*F*_1,90_ = 71.557, *p* < 0.001, *post hoc* tests: all *p* < 0.001), showing that pups that had been exposed to ELS had gained significantly less weight than STD-housed pups (**Figure 1C**). In addition, there was a small difference in bodyweight between the mouse lines (*F*_2,90_ = 2.846, *p* = 0.063, *post hoc* tests: HR vs. LR: *p* = 0.089, all other between-lines comparisons: *p* > 0.1). On P17, the main effect of housing condition remained significant (*F*_1,87_ = 20.396, *p* < 0.001, *post hoc* tests for ELS vs. STD: HR: *p* = 0.004, IR: *p* = 0.012, LR: *p* = 0.023) and a comparison between the three lines revealed that LR pups weighed more than HR pups (*F*_2,87_ = 3.339, *p* = 0.040, *post hoc* tests: HR vs. LR: *p* = 0.036, all other: *p* > 0.1) (**Figure 1D**). At weaning on P25, only the main effect of condition was significant (*F*_1,87_ = 18.181, *p* < 0.001, *post hoc* tests: HR: *p* = 0.032, IR: *p* = 0.007, LR: *p* = 0.016) (**Figure 1E**). During early adulthood, there was a clear differences in bodyweight between animals of the three lines, increasing from HR to IR to LR (*F*_2,127_ = 169.490, *p* < 0.001, *post hoc* tests: all *p* < 0.001), as well as a main effect of ELS exposure (*F*_1,127_ = 17.543, *p* < 0.001), and an interaction of line and condition (*F*_2,127_ = 5.317, *p* = 0.007). Further analysis specified that the ELS effect was significant in the HR (*p* < 0.001) and the LR mouse line (*p* = 0.026) (**Figure 1F**). During late adulthood, HR mice still weighed significantly less than animals of the other two lines (*F*_2,65_ = 54.141, *p* < 0.001, *post hoc* tests: HR vs. IR and LR: *p* < 0.001), but IR and LR mice no longer differed (IR vs. LR: *p* = 1.0). In addition, the effect of ELS housing was still significant (*F*_1,65_ = 5.331, *p* = 0.024), and *post hoc* tests showed a statistical trend in the LR line (*p* = 0.096) (**Figure 1G**).

### Results of the Behavioral and Cognitive Testing

#### Exploratory Behavior in the Open Field Test Was Not Affected by ELS

**FIGURE 2:**
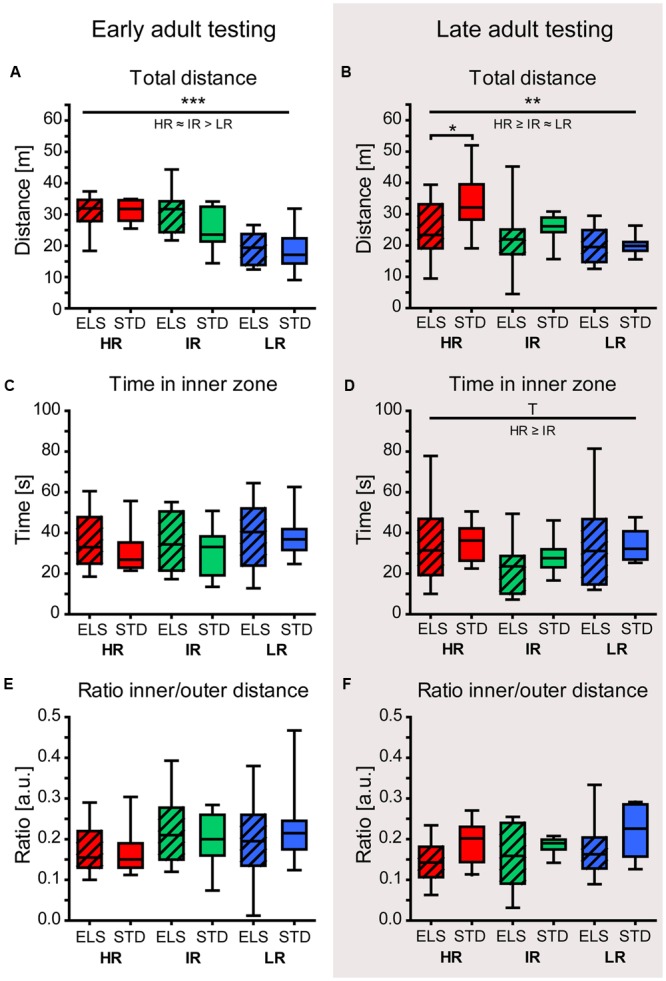
**Open field test (OFT) in early and late adulthood.** The behavior of HR, IR, and LR mice, raised in ELS or STD housing conditions was analyzed by univariate ANOVA, *N* = 10 per group. Data are presented as boxplots showing the median (horizontal line in the box), 25–75% (boxes) and 10–90% (whiskers). **(A)** The total distance traveled in the OFT in early adulthood differed significantly between the three mouse lines (*F*_2,54_ = 23.371, *p* < 0.001, *post hoc* tests: HR vs. IR: *p* = 0.409, HR vs. LR: *p* < 0.001, IR vs. LR: *p* < 0.001), but not between conditions (*F*_1,54_ = 1.219, *p* = 0.274). **(B)** The total distance traveled during the OFT in late adulthood showed a main effect of line (*F*_2,54_ = 7.462, *p* = 0.001, *post hoc* tests: HR vs. IR: *p* = 0.084, HR vs. LR: *p* = 0.001, IR vs. LR: *p* = 0.357), as well as a main effect of condition (*F*_1,54_ = 4.130, *p* = 0.047, HR: *p* = 0.019, IR: *p* = 0.299 and LR: *p* = 0.954). **(C)** In early adulthood, the time the animals spent in the inner zone of the OF was not affected by line (*F*_2,54_ = 0.980, *p* = 0.382) or condition (*F*_1,54_ = 0.635, *p* = 0.429). **(D)** In late adulthood, the time in the inner zone showed a trend for a main effect of line (*F*_2,54_ = 3.002, *p* = 0.058, *post hoc* tests: HR vs. IR: *p* = 0.055, HR vs. LR: *p* = 0.985, IR vs. LR: *p* = 0.459), but no effect of condition (*F*_1,54_ = 0.093, *p* = 0.762). **(E)** and **(F)** Both in early and late adulthood, the ratio of the inner to outer path length showed no significant effect of line (early: *F*_2,54_ = 1.642, *p* = 0.203; late: *F*_2,54_ = 0.576, *p* = 0.565) or condition (early: *F*_1,54_ = 0.006, *p* = 0.940; late: *F*_1,54_ = 2.320, *p* = 0.134). ^∗∗∗^*p* ≤ 0.001, ^∗∗^*p* ≤ 0.01, ^∗^*p* ≤ 0.05, ^T^*p* ≤ 0.1. Main effects of line are represented above a horizontal line above the graphs. The respective *post hoc* test statistics are indicated underneath the line with: </>, *p* ≤ 0.05; ≤/≥, *p* ≤ 0.1; ≈, *p* > 0.1. *Post hoc* statistics for main effects of condition and the interaction are presented above the appropriate boxes.

When animals were tested during early adulthood, the total distance traveled in the OF arena revealed a main effect of mouse line (*F*_2,54_ = 23.371, *p* < 0.001). Specifically, LR mice traveled shorter distances than HR and IR mice (*post hoc* tests: HR vs. IR: *p* = 0.409, HR vs. LR: *p* < 0.001, IR vs. LR: *p* < 0.001) (McIlwrick et al., 2016, also shown in **Figure 2A**), confirming previous findings in the SR mouse model (Touma et al., 2008; Heinzmann et al., 2014). ELS exposure had no effect on the animals’ locomotor activity in the OFT at this time point. When animals were tested during late adulthood, there was again a significant effect of mouse line on the total distance traveled (*F*_2,54_ = 7.462, *p* = 0.001, *post hoc* tests: HR vs. IR: *p* = 0.084, HR vs. LR: *p* = 0.001, IR vs. LR: *p* > 0.1) (**Figure 2B**). In addition, the analysis revealed a main effect of condition (*F*_1,54_ = 4.130, *p* = 0.047), and *post hoc* tests showed that ELS-exposed mice in the HR line tended to move around less than STD-housed HR mice (ELS vs. STD: HR: *p* = 0.019, IR and LR: *p* > 0.1).

At both time points (early and late adulthood), there was no indication of an effect of ELS on anxiety-related behavior (time in the inner zone: early adulthood: *F*_2,54_ = 0.635, *p* = 0.429, late adulthood: *F*_2,54_ = 0.093, *p* = 0.762) (**Figures 2C,D**). Animals tested in late adulthood showed a trend for a main effect of mouse line regarding the time spent in the inner zone of the OF (*F*_2,54_ = 3.002, *p* = 0.058, *post hoc* tests: HR vs. IR: *p* = 0.055, HR vs. LR and IR vs. LR: *p* > 0.1). This may, however, be related to the increased locomotor activity of HR mice in general. In line with this, at both time points, the ratio of the path length the animals traveled in the inner and outer zone showed no significant difference between the lines (early: *F*_2,54_ = 1.642, *p* = 0.203; late: *F*_2,54_ = 0.576, *p* = 0.565) or between conditions (early: *F*_1,54_ = 0.006, *p* = 0.940; late: *F*_1,54_ = 2.320, *p* = 0.134) (**Figures 2E,F**).

#### ELS Affects Spatial Learning in the Water Cross-Maze in HR and IR Animals

**FIGURE 3:**
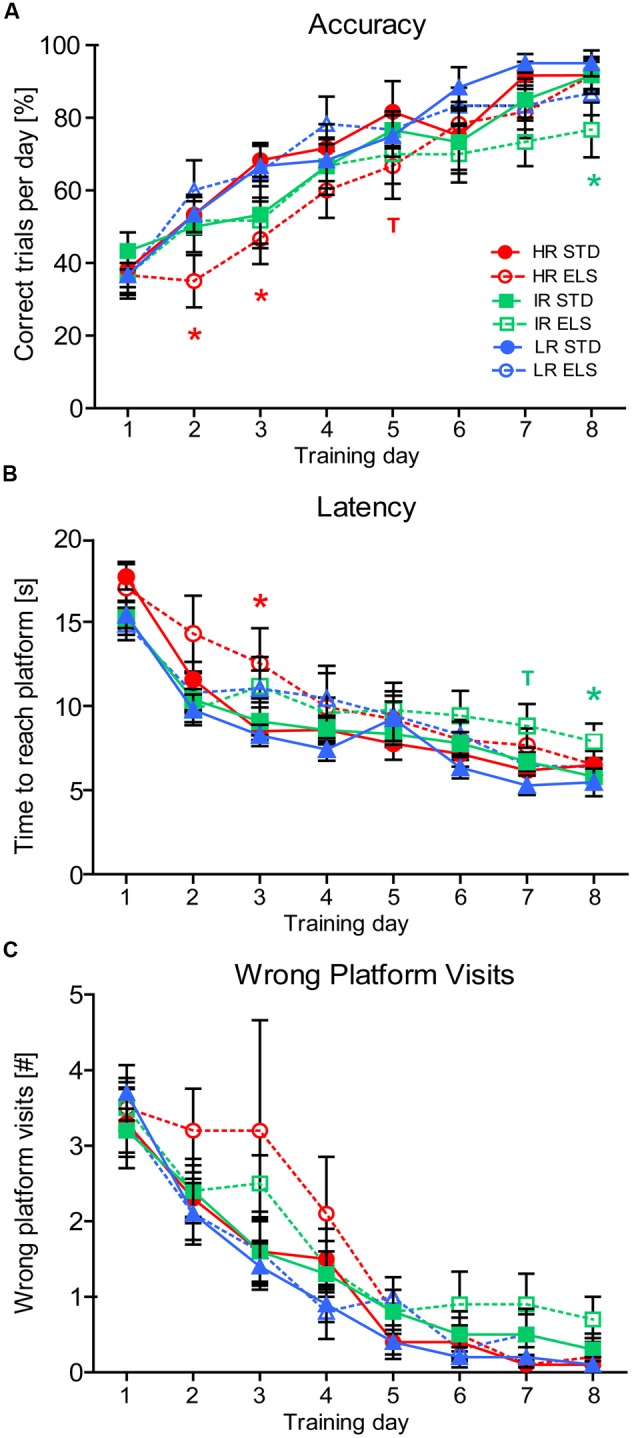
**Water Cross-Maze test.** Test performance of HR, IR, and LR mice, raised in ELS or STD housing conditions was analyzed by univariate ANOVA, *N* = 10 per group. Data was analyzed using repeated-measures ANOVA and is presented as line plots showing means and SEM (error bars). **(A)** The accuracy of the animals task performance showed a within-subjects main effect of training day (*F*_7,378_ = 62.794, *p* < 0.001), and a trend for a between-subjects main effect of condition (*F*_1,54_ = 2.882, *p* = 0.095), but no effect of line (*F*_2,54_ = 1.753, *p* = 0.183). Specifically, HR ELS mice were less accurate than HR STD mice on testing days 2, 3, and 5 (*p* = 0.040, *p* = 0.032, *p* = 0.094) and IR ELS mice were less accurate than IR STD mice on day 8 (*p* = 0.048). Overall pairwise comparisons showed a trend for poorer accuracy in the HR ELS compared to HR STD animals (*F*_1,54_ = 3.085, *p* = 0.085), but no difference in the IR and LR lines(*F*_1,54_ = 0.986, *p* = 0.325, and *F*_1,54_ = 0.036, *p* = 0.849). **(B)** The latency to reach the hidden platform showed a within-subjects main effect of training day (*F*_7,378_ = 85.502, *p* < 0.001), and a between-subjects main effect of condition (*F*_1,54_ = 4.123, *p* = 0.047), but no effect of line (*F*_2,54_ = 0.635, *p* = 0.534). Specifically, HR ELS mice had a higher latency than HR STD mice on testing day 3 (*p* = 0.032) and IR ELS had a higher latency than IR STD mice on days 7 and 8 (*p* = 0.060 and *p* = 0.040). Overall pairwise comparisons were not significant (HR: *F*_1,54_ = 1.562, *p* = 0.217, IR: *F*_1,54_ = 1.218, *p* = 0.275, LR: *F*_1,54_ = 1.353, *p* = 0.250). **(C)** The number of wrong platform visits showed a within-subjects main effect of training day (*F*_7,378_ = 60.502, *p* < 0.001), and a trend for a between-subjects main effect of condition (*F*_1,54_ = 3.376, *p* = 0.072), but no effect of line (*F*_2,54_ = 1.348, *p* = 0.268). No particular day showed significant differences between conditions, but overall pairwise comparisons revealed a trend for an increased number of wrong platform visits in the HR ELS compared to HR STD mice (*F*_1,54_ = 2.891, *p* = 0.095), but not in IR and LR animals IR: *F*_1,54_ = 1.188, *p* = 0.281, LR: *F*_1,54_ = 0.154, *p* = 0.696). ^∗∗∗^*p* ≤ 0.001, ^∗∗^*p* ≤ 0.01, ^∗^*p* ≤ 0.05; ^T^*p* ≤ 0.1. *Post hoc* statistics for main effects of condition and the interaction are presented above the appropriate data points of the line plot in the corresponding color.

To assess spatial learning and memory, we first evaluated each animal’s performance during the 8 days of training in the WCM test. A repeated-measures ANOVA confirmed that, overall, the animals in all six experimental groups improved their task performance over time, showing an increased accuracy (*F*_7,378_ = 62.794, *p* < 0.001) (**Figure 3A**), a decreased latency to reach the hidden platform (*F*_7,378_ = 85.502, *p* < 0.001) (**Figure 3B**), and a decreasing number of wrong platform visits (*F*_7,378_ = 60.502, *p* < 0.001) (**Figure 3C**). We next analyzed the data for between-group effects and found a trend for a main effect of condition on accuracy (*F*_1,54_ = 2.882, *p* = 0.095) (**Figure 3A**), a significant effect of condition on latency (*F*_1,54_ = 4.123, *p* = 0.047) (**Figure 3B**), as well as a trend for a main effect of condition on the number of wrong platform visits (*F*_1,54_ = 3.376, *p* = 0.072) (**Figure 3C**). *Post hoc* tests for the accuracy measures revealed that on training days 2, 3, and 5 HR ELS mice performed worse than HR STD mice (*p* = 0.040, *p* = 0.032, and *p* = 0.094, respectively), leading, overall, to a statistical trend for decreased accuracy in HR ELS compared to HR STD mice (*F*_1,54_ = 3.085, *p* = 0.085). IR ELS animals also had lower accuracy scores than IR STD animals on training day 8 (*p* = 0.048), but overall, the performance of IR ELS mice was not significantly different from IR STD animals. LR mice showed no significant effects of ELS on accuracy in this task. *Post hoc* tests for the latency to reach the platform showed that both HR and IR ELS mice had some deficits on different testing days (HR, day 3: *p* = 0.032; IR, days 7 and 8: *p* = 0.060 and *p* = 0.040), but over the course of the entire 8 days of testing, the difference in latency scores between ELS and STD mice was not significant in any of the three lines. *Post hoc* analysis of the number of wrong platform visits, overall, revealed that, although there were no significant differences on any particular training day, there was a trend for more platform errors in HR ELS compared to HR STD mice (*F*_1,54_ = 2.891, *p* = 0.095), but no difference between ELS and STD mice in the other two mouse lines. Together this data indicates ELS-induced deficiencies in the acquisition of hippocampus-dependent place learning and spatial navigation in the WCM mainly in HR mice, as well as similar but weaker effects in IR animals.

#### HR and LR Mice Show Divergent Effects of ELS on Spatial Memory in the Y-Maze Test

**FIGURE 4:**
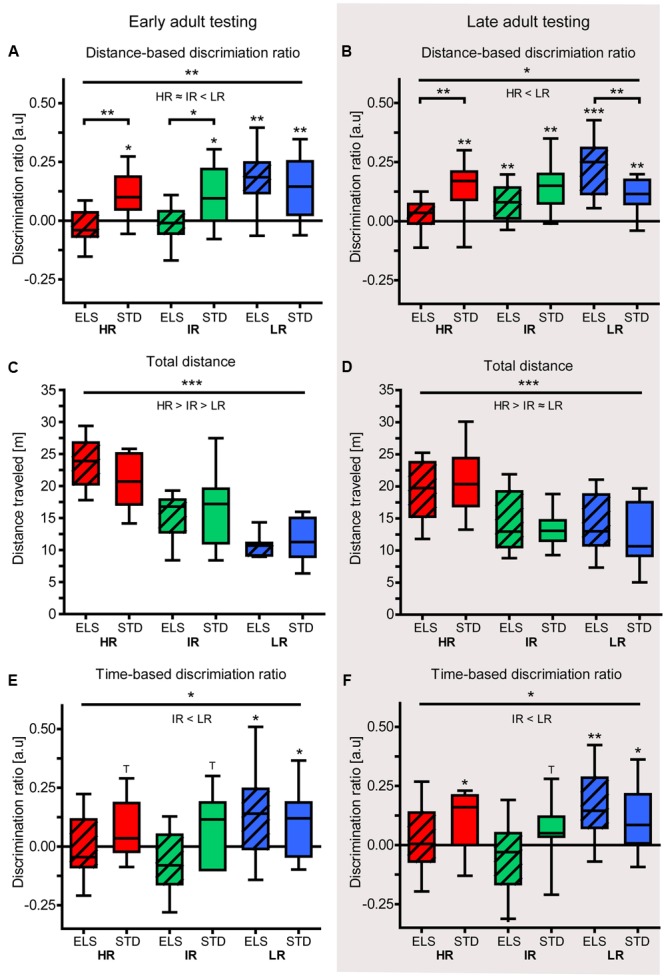
**Y-maze test in early and late adulthood.** Test performance of HR, IR, and LR mice, raised in ELS or STD housing conditions was analyzed by one-sample *t*-tests and univariate ANOVA, *N* = 9–10 per group. Data are presented as boxplots showing the median (horizontal line in the box), 25–75% (boxes) and 10–90% (whiskers). **(A)** In early adulthood, the discrimination ratio (based on distance) showed that LR animals, as well as HR STD and IR STD animals, discriminated between novel and familiar arms by traveling further distances in the novel arm, but HR ELS and IR ELS mice did not (HR ELS: *t*_9_ = -1.302, *p* = 0.113, HR STD: *t*_9_ = 3.255, *p* = 0.005, IR ELS: *t*_9_ = -0.412, *p* = 0.345, IR STD: *t*_9_ = 2.648, *p* = 0.014, LR ELS: *t*_9_ = 4.346, *p* = 0.001, LR STD: *t*_9_ = 3.487, *p* = 0.004). The ANOVA revealed a main effect of line (*F*_2,54_ = 7.785, *p* = 0.001, *post hoc* tests: HR vs. IR: *p* = 1.0, HR vs. LR: *p* = 0.002, IR vs. LR: *p* = 0.006), a main effect of condition (*F*_1,54_ = 6.828, *p* = 0.012), and an interaction of line and condition (*F*_1,54_ = 3.297, *p* = 0.045, *post hoc* tests: HR: *p* = 0.009, IR: *p* = 0.020 and LR: *p* = 0.564). **(B)** In late adulthood, the discrimination ratio (based on distance) showed that only HR ELS mice did not discriminate between novel and familiar arms (HR ELS: *t*_9_ = 1.134, *p* = 0.142, HR STD: *t*_8_ = 3.768, *p* = 0.003, IR ELS: *t*_9_ = 3.256, *p* = 0.005, IR STD: *t*_8_ = 4.362, *p* = 0.001, LR ELS: *t*_9_ = 6.119, *p* < 0.001, LR STD: *t*_9_ = 4.614, *p* = 0.001). The ANOVA showed a main effect of mouse line (*F*_2,52_ = 4.410, *p* = 0.017, *post hoc* tests: HR vs. IR: *p* = 0.940, HR vs. LR: *p* = 0.012, IR vs. LR: *p* = 0.157), and a significant interaction (*F*_2,52_ = 8.978, *p* > 0.001, *post hoc* tests: HR: *p* = 0.009, LR: *p* = 0.128, LR: *p* = 0.005). **(C)** The total distance travel by the animals in early adulthood in the Y-maze showed a main effect of line (*F*_2,54_ = 40.514, *p* < 0.001, *post hoc* test: all *p* ≤ 0.001), while condition had no effect (*F*_1,54_ = 0.114, *p* = 0.707). **(D)** The total distance travel by the animals in late adulthood in the Y-maze showed a main effect of line (*F*_2,52_ = 13.059, *p* < 0.001, *post hoc* test: HR vs. IR and vs. LR: *p* ≤ 0.001, IR vs. LR: *p* = 1.0), while condition had no effect (*F*_1,52_ = 0.737, *p* = 0.484). **(E)** In early adulthood, the discrimination ratio (based on time) showed that only LR mice discriminated between the novel and familiar arms by spending significantly more time in the novel arm (HR ELS: *t*_9_ = -0.258, *p* = 0.401, HR STD: *t*_9_ = 1.739, *p* = 0.058, IR ELS: *t*_9_ = -1.493, *p* = 0.085, IR STD: *t*_9_ = 1.754, *p* = 0.056, LR ELS: *t*_9_ = 2.361, *p* = 0.022, LR STD: *t*_9_ = 2.104, *p* = 0.033). The ANOVA revealed a main effect of line (*F*_2,54_ = 3.315, *p* = 0.044, *post hoc* tests: HR vs. IR: *p* = 1.0, HR vs. LR: *p* = 0.154, IR vs. LR: *p* = 0.058), but no effect of condition (*F*_1,54_ = 2.404, *p* = 0.127) and no interaction (*F*_2,54_ = 2.037, *p* = 0.140). **(F)** In late adulthood, the discrimination ratio (based on time) showed that LR mice (from both conditions), IR STD mice, and HR STD mice discriminated between novel and familiar arms by spending significantly more time in the novel arm, but not IR and HR ELS animals (HR ELS: *t*_9_ = 0.392, *p* = 0.352, HR STD: *t*_9_ = 1.905, *p* = 0.045, IR ELS: *t*_9_ = -0.939, *p* = 0.186, IR STD: *t*_9_ = 1.715, *p* = 0.060, LR ELS: *t*_9_ = 3.258, *p* = 0.005, LR STD: *t*_9_ = 2.394, *p* = 0.020). The ANOVA revealed only a significant main effect of line (*F*_2,54_ = 4.039, *p* = 0.023, *post hoc* tests: HR vs. IR: *p* = 1.0, HR vs. LR: *p* = 0.196, IR vs. LR: *p* = 0.022; main effect of condition: *F*_1,54_ = 1.118, *p* = 0.295, interaction: *F*_2,54_ = 1.884, *p* = 0.162). ^∗∗∗^*p* ≤ 0.001, ^∗∗^*p* ≤ 0.01, ^∗^*p* ≤ 0.05, ^T^*p* ≤ 0.1. Main effects of line are represented above a horizontal line above the graphs. The respective *post hoc* test statistics are indicated underneath the line with: </>, *p*≤0.05; ≤/≥, *p* ≤ 0.1; ≈, *p* > 0.1. *Post hoc* statistics for main effects of condition and the interaction are presented above the appropriate boxes.

The animals’ spatial memory performance was assessed using the Y-maze test. In early adulthood, the animals’ distance-based discrimination ratio revealed that LR animals differentiated between novel and familiar and more extensively explored the novel arm, compared to the familiar arms (one sample *t*-test against test value zero: LR ELS: *t*_9_ = 4.346, *p* = 0.001, LR STD: *t*_9_ = 3.487, *p* = 0.004) (**Figure 4A**). Similarly, HR and IR mice that had been raised in STD conditions also made this distinction by traveling longer distances in the novel than in the familiar arms (HR STD: *t*_9_ = 3.255, *p* = 0.005, IR STD: *t*_9_ = 2.648, *p* = 0.014). However, HR and IR mice that had been exposed to ELS showed no preference for the novel arm (HR ELS: *t*_9_ = -1.302, *p* = 0.113, IR ELS: *t*_9_ = -0.412, *p* = 0.345). A comparison between all six experimental groups revealed a main effect of line (*F*_2,54_ = 7.785, *p* = 0.001, *post hoc* tests: HR vs. IR: *p* > 0.1, HR vs. LR: *p* = 0.002, IR vs. LR: *p* = 0.006), a main effect of condition (*F*_1,54_ = 6.828, *p* = 0.012), and an interaction of line and condition (*F*_1,54_ = 3.297, *p* = 0.045, *post hoc* tests: HR: *p* = 0.009, IR: *p* = 0.020 and LR: *p* > 0.1).

In line with the results seen in young adult animals, the analysis of the Y-maze test in late adulthood revealed that, in terms of exploration distance, only HR ELS did not discriminate between the novel and the familiar arms (one sample *t*-test against test value zero: HR ELS: *t*_9_ = 1.134, *p* = 0.142, HR STD: *t*_8_ = 3.768, *p* = 0.003, IR ELS: *t*_9_ = 3.256, *p* = 0.005, IR STD: *t*_8_ = 4.362, *p* = 0.001, LR ELS: *t*_9_ = 6.119, *p* < 0.001, LR STD: *t*_9_ = 4.614, *p* = 0.001) (**Figure 4B**). In this cohort, one HR STD and one IR STD animal had to be excluded from the analysis due to difficulties in video tracking. The ANOVA comparing the performance of all six groups showed a main effect of mouse line (*F*_2,52_ = 4.410, *p* = 0.017, *post hoc* tests: HR vs. IR, and IR vs. LR: *p* > 0.1, HR vs. LR: *p* = 0.012), as well as a significant interaction of line and condition (*F*_2,52_ = 8.978, *p* < 0.001). *Post hoc* tests specified that HR ELS animals performed significantly worse than HR STD mice (*p* = 0.009), while LR ELS mice actually outperformed LR STD animals (*p* = 0.005).

Both at the early and the late adulthood time point, there was a main effect of mouse line on the total exploration distance in the Y-maze (early adulthood: *F*_2,54_ = 40.514, *p* < 0.001, *post hoc* test: all *p* ≤ 0.001; late adulthood: *F*_2,52_ = 13.059, *p* < 0.001, *post hoc* test: HR vs. IR and vs. LR: *p* ≤ 0.001, IR vs. LR: *p* = 1.0), showing that HR mice were more active than the other two lines (**Figures 4C,D**). Importantly, there was no difference in the total distance traveled by ELS and STD animals at both time points (early adulthood: *F*_1,54_ = 0.114, *p* = 0.737; late adulthood: *F*_1,52_ = 0.103, *p* = 0.750).

As a further measure of spatial memory performance, we analyzed the time the animals spent in the different areas of the maze during the test. In early adulthood, only LR mice discriminated between the novel and the familiar arms of the Y-maze in terms of the time they spent exploring the different arms (one sample *t*-test against test value zero: LR ELS: *t*_9_ = 2.361, *p* = 0.022, LR STD: *t*_9_ = 2.104, *p* = 0.033) (**Figure 4E**). IR STD and HR STD animals showed a statistical trend (IR STD: *t*_9_ = 1.754, *p* = 0.056, HR STD: *t*_9_ = 1.739, *p* = 0.058), but IR ELS and HR ELS animals failed to show this discrimination or spent more time exploring the familiar arms (IR ELS: *t*_9_ = -1.493, *p* = 0.085, HR ELS: *t*_9_ = -0.258, *p* = 0.401). When comparing the groups in a univariate ANOVA, a main effect of mouse line was confirmed (*F*_2,54_ = 3.315, *p* = 0.044, *post hoc* tests: HR vs. IR and vs. *p* > 0.1, IR vs. LR: *p* = 0.058), but condition did not affect this read-out of spatial memory performance.

Animals tested during late adulthood showed a similar pattern of results. Specifically, LR mice showed a significant preference for the novel arm (LR ELS: *t*_9_ = 3.258, *p* = 0.005, LR STD: *t*_9_ = 2.394, *p* = 0.020) (**Figure 4F**). In the HR and IR mouse lines, those animals that had been raised in STD conditions made a distinction between novel and familiar arms (HR STD: *t*_8_ = 1.905, *p* = 0.045, IR STD: *t*_8_ = 1.715, *p* = 0.060), while ELS-exposed animals did not (HR ELS: *t*_9_ = 0.392, *p* = 0.352, IR ELS: *t*_9_ = -0.939, *p* = 0.186). The ANOVA showed a main effect of mouse line (*F*_2,54_ = 4.039, *p* = 0.023, *post hoc* tests: HR vs. IR, and vs. LR: *p* > 0.1, IR vs. LR: *p* = 0.022), but no effect of condition.

To detect an effect of the animals’ age on their spatial memory performance in the Y-maze test, “age” was included as independent factor in a univariate ANOVA. The results showed a main effect of age on the animals’ performance in the distance-based discrimination ratio (*F*_1,106_ = 5.016, *p* = 0.027), but there was no significant difference between early and late adulthood performance in the time-based discrimination measure.

#### ELS Impairs Object Recognition in HR Animals

**FIGURE 5:**
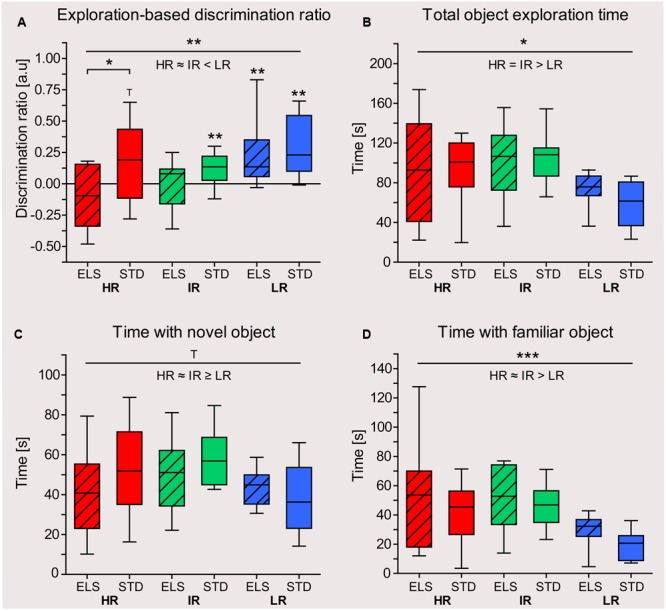
**Object recognition test in late adulthood.** Test performance of HR, IR, and LR mice, raised in ELS or STD housing conditions was analyzed using one-sample *t*-tests and univariate ANOVA, *N* = 10 per group. Data are presented as boxplots showing the median (horizontal line in the box), 25–75% (boxes) and 10–90% (whiskers). **(A)** The discrimination ratio, based on the exploration time animals spent with both objects, showed that IR STD, LR ELS, and LR STD mice preferentially explored the novel object, while HR STD mice showed a trend. IR ELS and HR ELS mice did not show a preference for the novel object or even showed a trend for preferring the familiar object (HR ELS: *t*_9_ = -1.499, *p* = 0.084, HR STD: *t*_8_ = 1.617, *p* = 0.073, IR ELS: *t*_9_ = 0.118, *p* = 0.454, IR STD: *t*_9_ = 2.943, *p* = 0.008, LR ELS: *t*_9_ = 2.834, *p* = 0.010, LR STD: *t*_8_ = 3.835, *p* = 0.003). The ANOVA showed a main effect of mouse line (*F*_2,52_ = 6.003, *p* = 0.005, *post hoc* tests: HR vs. IR: *p* = 0.1, HR vs. LR: *p* = 0.006, IR vs. LR: *p* = 0.025) and a main effect of condition (*F*_1,52_ = 6.925, *p* = 0.011, *post hoc* tests: HR ELS vs. STD: *p* = 0.011, IR *p* = 0.279, LR ELS vs. STD: *p* = 0.412). **(B)** The total exploration time animals spent with both objects showed a main effect of line (*F*_2,52_ = 6.663, *p* = 0.003, *post hoc* tests HR vs. IR: *p* = 1.0, HR vs. LR: *p* = 0.039, IR vs. LR: *p* = 0.003), but no effect of condition. **(C)** The time animals spent with the novel object showed a trend for a main effect of line (*F*_2,52_ = 2.532, *p* = 0.089, *post hoc* tests HR vs. IR: *p* = 0.526, HR vs. LR: *p* = 1.0, IR vs. LR: *p* = 0.094), but no effect of condition. **(D)** HR and IR mice spent more time exploring the familiar objects compared to LR animals (*F*_2,52_ = 7.884, *p* = 0.001, *post hoc* tests HR vs. IR: *p* = 0.1, HR vs. LR: *p* = 0.005, IR vs. LR: *p* = 0.002), and there was a trend for a main effect of condition (*F*_1,52_ = 3.191, *p* = 0.080, *post hoc* tests: HR ELS vs. STD: *p* = 0.156, IR *p* = 0.543, LR ELS vs. STD: *p* = 0.307). ^∗∗∗^*p* ≤ 0.001, ^∗∗^*p* ≤ 0.01, ^∗^*p* ≤ 0.05, ^T^*p* ≤ 0.1. Main effects of line are represented above a horizontal line above the graphs. The respective *post hoc* test statistics are indicated underneath the line with: </>, *p* ≤ 0.05; ≤/≥, *p* ≤ 0.1; ≈, *p* > 0.1. *Post hoc* statistics for main effects of condition and the interaction are presented above the appropriate boxes.

To detect whether the animals were able to distinguish between the previously encountered and the novel object, a discrimination ratio was calculated for each animal, as described in the section “Behavioral and Cognitive Testing.” One HR STD and one LR STD animal had to be excluded from the analysis, because they did not reach the criterion of exploring both objects for at least 5 s during the familiarization phase. Using one-sample *t*-tests (against test value 0), the analysis showed that both HR ELS and IR ELS animals did not spend more time exploring the novel object (HR ELS mice actually showed a trend for favoring the familiar object) (one-sided *t*-tests: HR ELS: *t*_9_ = -1.499, *p* = 0.084, IR ELS: *t*_9_ = 0.118, *p* = 0.454) (**Figure 5A**). HR STD mice showed a trend for positive object discrimination (HR STD: *t*_8_ = 1.617, *p* = 0.073), and animals from the IR STD group, as well as LR ELS and LR STD all spent significantly more time investigating the novel object (IR STD: *t*_9_ = 2.943, *p* = 0.008, LR ELS: *t*_9_ = 2.834, *p* = 0.010, LR STD: *t*_8_ = 3.835, *p* = 0.003), thus showing they remembered the previously encountered familiar object. Comparisons between the experimental groups using a univariate ANOVA revealed a significant main effect of line (*F*_2,52_ = 6.003, *p* = 0.005), showing that LR animals performed significantly better in this task than HR (*p* = 0.006) and IR mice (*p* = 0.025). In addition, the analysis showed a main effect of condition (*F*_1,52_ = 6.925, *p* = 0.011) and *post hoc* tests specified that this effect was only significant in the HR mouse line (*p* = 0.011), i.e., overall, HR ELS mice performed significantly worse that HR STD animals.

During the testing phase, LR animals spent overall less time exploring both objects than IR and HR mice (*F*_2,52_ = 6.663, *p* = 0.003, *post hoc* tests HR vs. IR: *p* = 0.1, HR vs. LR: *p* = 0.039, IR vs. LR: *p* = 0.003), but there was no difference between ELS and STD-housed animals (**Figure 5B**). A similar pattern was seen when examining the animals’ exploration of each object separately (**Figures 5C,D**).

### HR Animals Show Increased Stress Reactivity after ELS Exposure

**FIGURE 6:**
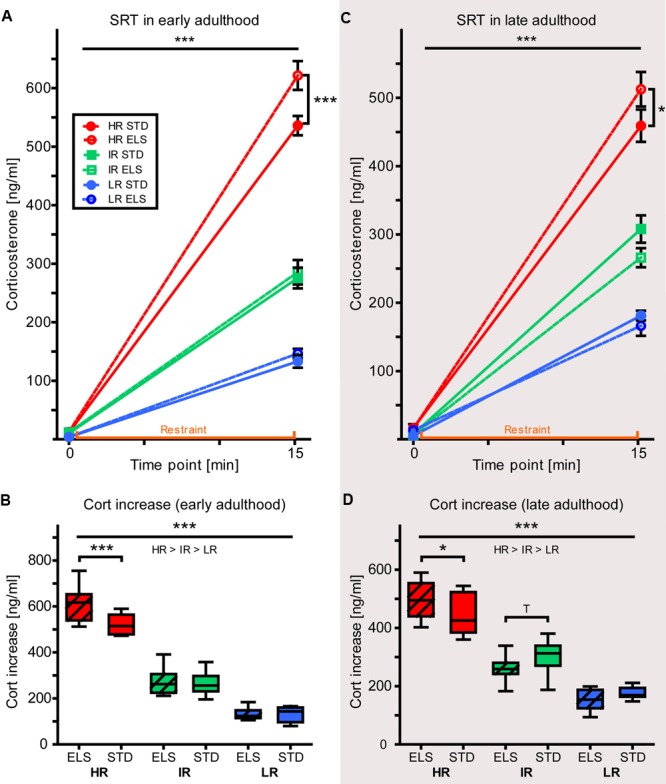
**Stress reactivity.** Corticosterone concentrations measured in the plasma of HR, IR, and LR mice, raised in ELS or STD housing conditions, collected during the SRT was analyzed using repeated-measured and univariate ANOVA, *N* = 8–10 per group. Data is presented as line plots showing means and SEM (error bars) and as boxplots showing the median (horizontal line in the box), 25–75% (boxes) and 10–90% (whiskers). **(A)** In early adulthood, there was a significant effect of time point (initial vs. response) on the plasma corticosterone concentration (within-subjects effect: *F*_1,54_ = 1459.996, *p* < 0.001). The initial corticosterone concentration showed a main effect of line (*F*_2,54_ = 6.253, *p* = 0.004, *post hoc* tests: HR vs. IR: *p* = 1.0, HR vs. LR: *p* = 0.007, IR vs. LR: *p* = 0.015), but no effect of condition (*F*_1,54_ = 0.148, *p* = 0.702). The response levels of corticosterone showed a main effect of mouse line (*F*_2,54_ = 214.164, *p* < 0.001, *post hoc* tests: all *p* < 0.001), as well as a main effect of condition (*F*_1,54_ = 5.675, *p* = 0.021), and an interaction of line and condition (*F*_2,54_ = 4.232, *p* = 0.020, *post hoc* tests, ELS vs. STD: HR: *p* < 0.001, IR: *p* = 0.742, LR: *p* = 0.857). **(B)** The increase in plasma corticosterone levels in response to 15-min restraint showed a main effect of line (*F*_2,54_ = 223.319, *p* < 0.001, *post hoc* tests: all *p* < 0.001), a main effect of condition (*F*_1,54_ = 6.022, *p* = 0.017), as well as an interaction of line and condition (*F*_2,54_ = 6.243, *p* = 0.016, *post hoc* tests ELS vs. STD: HR: *p* < 0.001, IR: *p* = 0.776, LR: *p* = 0.949). **(C)** In late adulthood, there was a significant effect of time point (initial vs. response) on the plasma corticosterone concentration (within-subjects effect: *F*_1,53_ = 1957.027, *p* < 0.001). The initial corticosterone concentration showed a trend for a main effect of mouse line (*F*_2,53_ = 3.023, *p* = 0.057, *post hoc* tests: HR vs. IR: *p* = 0.063, HR vs. LR: *p* = 0.278, IR vs. LR: *p* = 1.0), but no effect of condition (*F*_1,53_ = 0.381, *p* = 0.540). The response levels of corticosterone showed a main effect of mouse line (*F*_2,54_ = 140.272, *p* < 0.001, *post hoc* tests: all *p* < 0.001), and interaction of line and condition (*F*_2,53_ = 3.468, *p* = 0.038, *post hoc* tests, ELS vs. STD: HR: *p* = 0.048, IR: *p* = 0.118, LR: *p* = 0.580). **(D)** The increase in plasma corticosterone levels in response to 15-min restraint showed a main effect of line (*F*_2,53_ = 166189, *p* < 0.001, *post hoc* tests: all *p* < 0.001), and an interaction of line and condition (*F*_2,53_ = 4.544, *p* = 0.015, *post hoc* tests, ELS vs. STD: HR: *p* = 0.026, IR: *p* = 0.0.092, LR: *p* = 0.344). ^∗∗∗^*p* ≤ 0.001, ^∗∗^*p* ≤ 0.01, ^∗^*p* ≤ 0.05, ^T^p ≤ 0.1. Main effects of line are represented above a horizontal line above the graphs. The respective *post hoc* test statistics are indicated underneath the line with: </>, *p* ≤ 0.05; ≤/≥, *p* ≤ 0.1; ≈, *p* > 0.1. *Post hoc* statistics for main effects of condition and the interaction are presented above or next to the appropriate boxes.

The absolute levels of plasma CORT concentration before (initial) and after (response) 15 min of restraint were analyzed using a repeated-measures ANOVA. In early adulthood, the data showed a strong effect of time point (initial vs. response), confirming a significant rise in plasma CORT levels in response to the stressor in animals of all three lines (within-subjects effect: *F*_1,54_ = 1459.996, *p* < 0.001) (**Figure 6A**). The initial CORT levels (taken within 2 min after the first disturbance of the animals’ cage) showed that HR and IR mice had higher baseline CORT levels than LR mice (*F*_2,54_ = 6.253, *p* = 0.004, *post hoc* tests: HR vs. IR: *p* = 1.0, HR vs. LR: *p* = 0.007, IR vs. LR: *p* = 0.015), but there was no difference between conditions (*F*_1,54_ = 0.148, *p* = 0.702). In the response levels measured after restraint, the main effect of mouse line was exacerbated, with evident differences in CORT concentrations between all three lines (*F*_2,54_ = 214.164, *p* < 0.001, *post hoc* tests: all *p* < 0.001). In addition, the analysis revealed a main effect of condition (*F*_1,54_ = 5.675, *p* = 0.021), as well as an interaction of line and condition (*F*_2,54_ = 4.232, *p* = 0.020), and *post hoc* comparisons showed that HR ELS mice had significantly higher plasma CORT levels in response to the stressor than HR STD mice (*p* < 0.001), while no other mouse line showed this effect of ELS.

As there had been differences between the groups at baseline, we also analyzed the CORT increase (reaction CORT minus initial CORT level) and this measure confirmed the main effect of mouse line (*F*_2,54_ = 223.319, *p* < 0.001, *post hoc* tests: all *p* < 0.001) (**Figure 6B**). In addition, the increase in plasma CORT concentration also revealed a main effect of condition (*F*_1,54_ = 6.022, *p* = 0.017), as well as an interaction of line and condition (*F*_2,54_ = 6.243, *p* = 0.016), showing that HR ELS mice had a significantly higher increase in CORT levels than HR STD mice (*p* < 0.001), while IR and LR mice did not show this effect of ELS exposure on stress reactivity (*p* = 0.776 and *p* = 0.949, respectively).

The SRT carried out in mice during late adulthood matched our earlier results (**Figure 6C**). Again, there was a strong effect of time point (initial vs. response) (*F*_1,53_ = 1957.027, *p* < 0.001) and the initial CORT values showed a strong trend for a main effect of line (*F*_2,53_ = 3.023, *p* = 0.057, *post hoc* tests: HR vs. IR: *p* = 0.063, HR vs. LR: *p* = 0.309 IR vs. LR: *p* = 1.0), but no effect of condition (*F*_1,53_ = 0.381, *p* = 0.540). The difference between the three lines became highly significant after 15 min of restraint (*F*_2,53_ = 140.272, *p* < 0.001, *post hoc* tests: all *p* < 0.001), when, in addition, there was also an interaction of line and condition (*F*_2,53_ = 3.468, *p* = 0.038). Specifically, ELS-exposed HR mice showed a significantly stronger CORT response than STD-raised HR mice (*p* = 0.048), while IR and LR mice seemed more resilient to this early-life programming of stress reactivity. The analysis of the increase in plasma CORT levels confirmed a main effect of mouse line (*F*_2,53_ = 166.189, *p* < 0.001, *post hoc* tests: all *p* < 0.001), and the interaction of line and condition (*F*_2,53_ = 4.544, *p* = 0.015, *post hoc* tests: HR: *p* = 0.026, IR: *p* = 0.092, LR: *p* = 0.344) (**Figure 6D**).

### ELS Is Associated with Long-Term Changes in Hippocampal Gene Expression

#### *Bdnf* Is Downregulated in HR Mice after ELS

**FIGURE 7:**
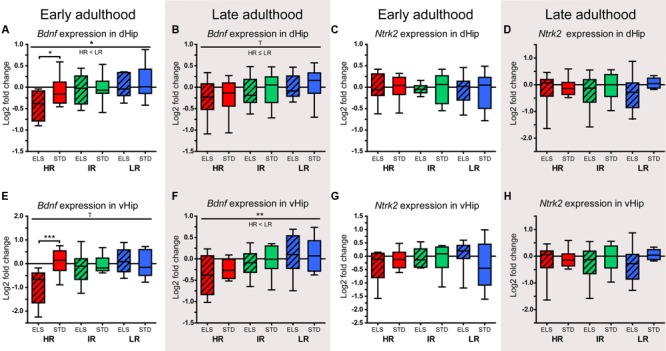
**Relative expression levels of *Bdnf* and *Ntrk2*.** The relative expression levels of the genes coding for the neurotrophin BDNF (*Bdnf*) and its receptor TRKB (*Ntrk2*) were measured in selected brain regions of HR, IR, and LR mice, raised in ELS or STD housing conditions, at an early and a late time point in adulthood. The data was normalized to the IR STD animals and analyzed using two-way ANOVAs, and is presented as boxplots showing the median (horizontal line in the box), 25–75% (boxes) and 10–90% (whiskers), *N* = 8–11 per group. **(A)** In early adulthood, the expression of *Bndf* in the dHip showed a main effect of line (*F*_1,54_ = 4,091, *p* = 0.022, *post hoc* tests: HR vs. IR: *p* = 0.301, HR vs. LR: *p* = 0.038 IR vs. LR: *p* = 1.0), as well as a trend for a main effect of condition (*F*_1,54_ = 3.177, *p* = 0.080, *post hoc* tests ELS vs. STD: HR: *p* = 0.031, IR: *p* = 0.972, LR: *p* = 0.442). **(B)** In late adulthood, the expression of *Bndf* in the dHip showed a trend for a main effect of line (*F*_2,52_ = 2.651, *p* = 0.080, *post hoc* tests: HR vs. IR: *p* = 0.615, HR vs. LR: *p* = 0.080, IR vs. LR: *p* = 0.931). **(C)** and **(D)** In early adulthood and in late adulthood, there was no effect of line of condition on the expression of *Ntrk2* in the dHip. **(E)** In early adulthood, the expression of *Bndf* in the vHip showed a trend for main effect of line (*F*_2,52_ = 2.993, *p* = 0.059, *post hoc* test: all *p* > 0.1), a main effect of condition (*F*_1,52_ = 4.513, *p* = 0.038), and an interaction of line and condition (*F*_2,52_ = 5.677, *p* = 0.006, *post hoc* tests ELS vs. STD: HR: *p* > 0.001, IR: *p* = 0.649, LR: *p* = 0.492). **(F)** In late adulthood, the expression of *Bndf* in the vHip showed a main effect of line (*F*_2,54_ = 5.218, *p* = 0.008, *post hoc* tests: HR vs. IR: *p* = 0.174, HR vs. LR: *p* = 0.007, IR vs. LR: *p* = 0.628). **(G)** and **(H)** In early adulthood and in late adulthood, there was no effect of line of condition on the expression of *Ntrk2* in the vHip. ^∗∗∗^*p* ≤ 0.001, ^∗∗^*p* ≤ 0.01, ^∗^*p* ≤ 0.05, ^T^*p* ≤ 0.1. Main effects of line are represented above a horizontal line above the graphs. The respective *post hoc* test statistics are indicated underneath the line with: </>, *p* ≤ 0.05; ≤/≥, *p* ≤ 0.1; ≈, *p* > 0.1. *Post hoc* statistics for main effects of condition and the interaction are presented above the appropriate boxes.

The analysis of gene expression in the dHip of animals sacrificed during early adulthood showed that *Bdnf* was downregulated in HR mice that had been exposed to ELS compared to STD-raised HR mice (*p* = 0.031) (**Figure 7A**). In addition, there was a main effect of line (*F*_1,54_ = 4,091, *p* = 0.022), showing that, overall, HR mice had lower *Bdnf* expression levels than LR animals (*p* = 0.038). When the expression levels of *Bdnf* were measured in animals sacrificed during late adulthood, the data showed a statistical trend in the same direction (*F*_2,52_ = 2.651, *p* = 0.080, *post hoc* tests: HR vs. LR: *p* = 0.080) (**Figure 7B**). Interestingly, the difference between HR ELS and STD mice was not present in the older animals; rather, at this later time point, the *Bdnf* expression levels in HR STD animals resembled those measured in HR ELS mice. The BDNF receptor coding gene *Ntrk2* showed no significant differences in expression between lines or conditions at either of the two time points in the dHip (**Figures 7C,D**).

Mirroring the pattern seen in the dHip, the levels of *Bdnf* in the vHip during early adulthood were significantly downregulated in HR ELS compared to HR STD mice (*p* > 0.001) (**Figure 7E**). In addition, the data showed a strong statistical trend for a main effect of line (*F*_2,52_ = 2.993, *p* = 0.059), a significant main effect of condition (*F*_1,52_ = 4.513, *p* = 0.038), and a significant interaction of line and condition (*F*_2,52_ = 5.677, *p* = 0.006). As seen in the dHip, the difference in *Bdnf* levels between HR ELS and STD mice was no longer significant when gene expression in the vHip was measured in late adulthood. Again, this seemed to be mainly due to a downward shift in the expression levels in HR STD animals (**Figure 7F**). There was a clear main effect of line (*F*_2,54_ = 5.218, *p* = 0.008), and, overall, HR animals had lower *Bdnf* levels than LR mice (*p* = 0.007), while there was no effect of condition. As in the dHip, the expression of *Ntrk2* in the vHip showed no significant effect of line or condition both in early and late adulthood (**Figures 7G,H**).

#### ELS Interacts with Genetic Predisposition in the Regulation of *Crh* Transcripts

**FIGURE 8:**
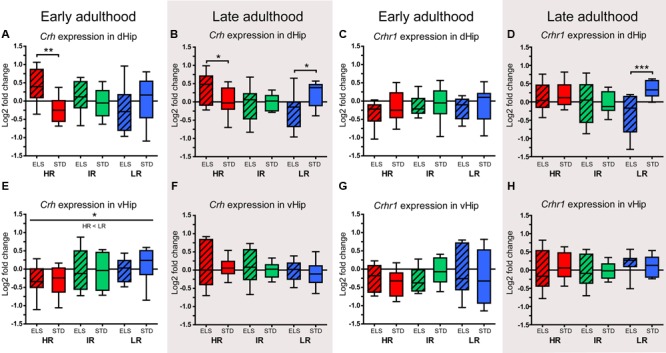
**Relative expression levels of *Crh* and *Crhr1.*** The relative expression levels of the genes coding for the neuropeptide CRH (*Crh*) and its receptor CRH-R1 (*Crhr1*) were measured in selected brain regions of HR, IR, and LR mice, raised in ELS or STD housing conditions, at an early and a late time point in adulthood. The data was normalized to the IR STD animals and analyzed using two-way ANOVAs, and is presented as boxplots showing the median (horizontal line in the box), 25–75% (boxes) and 10–90% (whiskers), *N* = 8–11 per group. **(A)** In early adulthood, the expression of *Crh* in the dHip showed an interaction of line and condition (*F*_2,48_ = 4.358, *p* = 0.018, *post hoc* tests ELS vs. STD: HR: *p* = 0.008, IR: *p* = 0.556, LR: *p* = 0.170). **(B)** In late adulthood, the expression of *Crh* in the dHip showed an interaction of line and condition (*F*_2,52_ = 5.247, *p* = 0.009, *post hoc* tests ELS vs. STD: HR: *p* = 0.047, IR: *p* = 0.743, LR: *p* = 0.015). **(C)** In early adulthood, the expression of *Crhr1* in the dHip showed no effect of line of condition. **(D)** In late adulthood, the expression of *Crhr1* in the dHip showed a main effect of condition (*F*_1,50_ = 4.798, *p* = 0.033) and an interaction of line and condition (*F*_2,50_ = 3.487, *p* = 0.038, *post hoc* tests ELS vs. STD: HR: *p* = 0.661, IR: *p* = 0.949, LR: *p* = 0.001). **(E)** In early adulthood, the expression of *Crh* in the vHip showed a main effect of line (*F*_1,53_ = 3.782, *p* = 0.029, *post hoc* tests: HR vs. IR: *p* = 0.162, HR vs. LR: *p* = 0.029, IR vs. LR: *p* = 1.0). **(F)** In late adulthood, the expression of *Crh* in the vHip showed no effect of line or condition. **(G)** and **(H)** In early and late adulthood, the expression of *Crhr1* in the vHip showed no effect of line or condition. ^∗∗∗^*p* ≤ 0.001, ^∗∗^*p* ≤ 0.01, ^∗^*p* ≤ 0.05, ^T^*p* ≤ 0.1. Main effects of line are represented above a horizontal line above the graphs. The respective *post hoc* test statistics are indicated underneath the line with: </>, *p* ≤ 0.05; ≤/≥, *p* ≤ 0.1; ≈, *p* > 0.1. *Post hoc* statistics for main effects of condition and the interaction are presented above the appropriate boxes.

In early adulthood, the expression of *Crh* in the dHip was affected by an interaction of line and condition (*F*_2,48_ = 4.358, *p* = 0.018). *Post hoc* analysis showed that HR ELS mice had higher *Crh* levels than HR STD animals (*p* = 0.008) (McIlwrick et al., 2016, also shown in **Figure 8A**). When measured in late adulthood, the same interaction was detected (*F*_2,52_ = 5.247, *p* = 0.009) and further analysis revealed that, as before, HR ELS mice had significantly higher *Crh* levels than HR STD mice (*p* = 0.047) and that the opposite was true in LR animals (*p* = 0.015), i.e., LR ELS animals displayed reduced *Crh* expression compared to LR STD mice (**Figure 8B**). The expression of the CRH-R1 gene did not differ significantly between lines and conditions in early adulthood (McIlwrick et al., 2016, also shown in **Figure 8C**). However, in late adulthood, the data showed an interaction of line and condition (*F*_2,50_ = 3.487, *p* = 0.038), with *post hoc* tests specifying that *Crhr1* levels were significantly reduced in LR ELS compare to LR STD mice (*p* = 0.001) (**Figure 8D**).

In the vHip, there was a main effect of line on the expression of *Crh* in early adulthood (*F*_1,53_ = 3.782, *p* = 0.029, *post hoc* tests: HR vs. LR: *p* = 0.029) (**Figure 8E**), but this effect was not observed in the later adulthood samples (**Figure 8F**). The expression of *Crhr1* in the vHip showed no significant changes associated with line or condition in early or late adulthood (**Figures 8G,H**).

### *Bdnf* and *Crh* Expression in the Dorsal Hippocampus are Associated with Differences in Cognitive Performance

The relationship between the animals’ cognitive test performance in late adulthood and the expression of *Bdnf, Crh*, and *Crhr1* in their dorsal and ventral hippocampus was investigated using the Pearson’s correlation coefficient. Our analyses revealed a significant positive correlation between *Bdnf* expression in the dorsal hippocampus and cognitive performance in the object recognition test (*r* = 0.265, *p* = 0.048). In addition, dorsal hippocampal *Bdnf* expression showed a strong statistical trend for a positive association with the mean performance in both cognitive tests [(discrimination ratio in the Y-maze test + discrimination ratio in the Object recognition test)/2] (*r* = 0.258, *p* = 0.059). On the other hand, the expression of *Crh* in the dorsal hippocampus showed a significant negative correlation with cognitive performance in the Y-maze test (*r* = 0.329, *p* = 0.017).

## Discussion

Here, we confirm previous findings showing that ELS produces late-onset and long-lasting effects on cognitive function (Brunson et al., 2005; Mehta and Schmauss, 2011; Gould et al., 2012). Our results further reveal that these effects differ between individuals, contingent with their innate stress reactivity (high vs. low). The inherited predisposition is therefore centrally involved in shaping the cognitive phenotype after ELS, and channeling the consequences at the level of neuroendocrine regulation and gene expression. Below, we discuss and integrate the key findings of the presented experiments in the light of current research regarding the complex interaction of genes and environment.

### Bodyweight and Behavior

Exposure to the ELS paradigm caused a substantial delay in bodyweight development in pups of all three SR mouse lines, evidenced by reduced bodyweight gain from P2 to P9 in litters raised in ELS conditions (**Figure 1C**). This main effect of ELS reflects a pronounced impact of the limited nesting and bedding material paradigm on the animals’ physiology and confirms previous findings in the SR mouse model, as well as in other rodent models (Gilles et al., 1996; Avishai-Eliner et al., 2008; Rice et al., 2008; Naninck et al., 2015; Bath et al., 2016; McIlwrick et al., 2016). The difference in bodyweight remained significant all throughout development into early and late adulthood in the HR and the LR lines, similar to observations by others (Bath et al., 2016). Animals in the IR ELS group matched their STD housed control group by early adulthood (**Figures 1D–G**). However, in a previous study in our animal model (McIlwrick et al., 2016), and as reported by others (Rice et al., 2008), no weight differences were observed between ELS and STD mice by early adulthood. The lasting effects of ELS on bodyweight will need to be investigated in future studies to allow better understanding of the circumstances under which long-term metabolic changes occur.

In line with previous studies using the limited nesting material paradigm or maternal separation (Brunson et al., 2005; Millstein and Holmes, 2007; Rice et al., 2008), ELS did not affect anxiety-related behavior in the adult offspring (**Figures 2C–F**). In general, the OFT results showed that HR mice were more active than LR mice, a phenotype which was present at both measurement time points (**Figures 2A,B**) and confirms earlier studies (Touma et al., 2008; Heinzmann et al., 2014). While there was an indication for reduced locomotor activity in aged HR ELS mice (**Figure 2B**), this finding was not confirmed in a slightly different testing set-up (**Figure 4D**), pointing toward a context-specific, rather than a general effect of ELS.

### Cognitive Function

Cognitive function was clearly influenced by ELS in the SR mouse model. However, the three lines were not equally vulnerable to the deleterious effects of early-life adversity. HR ELS animals showed the most pronounced phenotype in terms of cognitive impairments, emerging in early adulthood [indications for reduced spatial learning performance (**Figure 3**) and significantly impaired place memory (**Figure 4A**)] and lasting into older age [impaired place memory (**Figure 4B**) and impaired object memory (**Figure 5A**)]. IR ELS animals showed a similar effect in early adulthood [partially reduced spatial learning (**Figure 3**) and impaired place memory (**Figure 4A**)], but appeared to recover with increasing age [intact place memory (**Figure 4B**) and no significant difference in object memory (**Figure 5A**)]. In contrast, LR ELS animals showed no indication of cognitive deficits in early adulthood [normal spatial learning (**Figure 3**) and good place memory (**Figure 4A**)], and there was even some evidence for improved cognitive function in aged ELS-exposed LR mice compared to STD animals (**Figure 4B**). These results highlight that the consequences of ELS on cognitive function depend very much on the inherited predisposition of the individual. Previous studies in the SR mouse model have provided evidence for deficits in cognitive function in HR animals (Knapman et al., 2010a,b) and have linked this to reduced hippocampal activity and neuronal integrity (Knapman et al., 2012). The proposed mechanism underlying this phenotype in HR mice is a cumulative neurotoxic effect of glucocorticoids, as lifetime exposure to elevated stress hormones can give rise to progressive deficits in learning and memory (Hibberd et al., 2000; Sapolsky et al., 2000; Lupien et al., 2009). Our new data complements these findings by showing that the cognitive deficits observed in HR mice become exacerbated by ELS exposure. In a set of studies, Brunson et al. demonstrated that ELS can set off a cascade of structural and functional changes in different subfields of the hippocampus, including aberrant mossy fiber expansion, impaired long-term potentiation (LTP) and dendritic atrophy, which contribute to several cognitive impairments emerging with increased age (Brunson et al., 2005). We suggest that these same mechanisms may be acting in HR mice and that they may become increasingly detrimental through the additional sensitization of the HPA axis set in motion by the exposure to ELS.

### Stress Reactivity

To verify the central role of HPA axis programming in the effects of ELS, the animals’ stress reactivity was tested at two time points. Both in early and in late adulthood, the results showed that HR ELS mice had an increased stress reactivity compared to STD-raised HR mice (**Figure 6**). This ELS-induced augmentation in CORT release supports the hypothesis that the cognitive deficits displayed by HR ELS animals are due to excessive, cumulative glucocorticoid exposure and its adverse downstream consequences in stress sensitive regions of the brain, such as the hippocampus. Earlier work from our group demonstrated that directly after a week-long period of ELS exposure HR ELS pups had elevated basal CORT levels, which normalized by the age of weaning and remained low in adulthood (McIlwrick et al., 2016). In the present study, we again assessed the CORT levels in adult animals and found no differences between HR ELS and STD mice at baseline. The stress-induced CORT levels, however, did show a significant impact of ELS, replicating our previous results. This gradual shift from elevated basal CORT levels in pups to enhanced stress reactivity in adulthood suggests that a disruption of the HPA axis suppression during the stress-hyporesponsive period (SHRP) led to changes in the neuroendocrine programming of stress reactivity in these animals. During the SHRP, lasting from P2–P12 in mice, moderate stressors fail to elicit a measurable physiological stress response in the pups, due to a desensitization at all levels of the HPA axis (Sapolsky and Meaney, 1986). The suppression of the pups’ stress reactivity is tightly controlled by maternal care and can only be disrupted by severe stressors, such as removal of the dam (Levine, 2002; Schmidt et al., 2003). The SHRP coincides with a critical period of postnatal brain development, and its evolutionary purpose is most likely to minimize the damaging effects of glucocorticoids on the developing brain (Sapolsky and Meaney, 1986). In HR mice, the fragmented maternal care induced by the ELS paradigm was apparently sufficient to disrupt the suppression of the stress response system, leading to elevated basal CORT levels during the SHRP. As the GR-mediated negative feedback loop is not yet functioning in pups at this young age (Meaney et al., 1985), the CORT levels remained elevated with the potential to interfere with neuroendocrine receptor expression and to activate epigenetic processes in the developing brain. Once the negative feedback loop became functionally instantiated, basal CORT levels of HR ELS pups dropped to normal levels, while lasting epigenetic programming effects on the neuroendocrine system may have led to the augmented stress reactivity measured in adult HR ELS animals.

In contrast, adult LR mice showed no differences in their stress response associated with ELS rearing conditions, again replicating earlier results (McIlwrick et al., 2016). However, we noted that the baseline CORT levels of LR ELS mice increased significantly from early to late adulthood (pairwise comparisons LR ELS early vs. late adulthood: *p* = 0.031), and overall, LR mice displayed a rise in their stress response CORT levels in late adulthood (pairwise comparisons LR early vs. late adulthood: *p* = 0.051). To date, only some acute (pre-weaning) and no lasting effects of ELS exposure have been reported in LR animals (McIlwrick et al., 2016). Our new data now suggests that there are indeed some lasting consequences, but that these only appear with a late adulthood onset. Strikingly, the ELS-induced effects in LR mice, while not being very pronounced, seem to be rather favorable in nature: aged LR ELS animals showed signs of improved cognitive function and had slightly raised baseline CORT levels compared to LR STD mice. Since the effects of stress hormones have an inverted U-shaped relationship to cognition (de Kloet et al., 1999; Mateo, 2008; Sapolsky, 2015) and LR mice usually have a very low baseline HPA axis tone (Touma et al., 2008, 2009), a small increase in baseline activation may convey some beneficial aspects for attention and behavioral reactivity, by increasing the relative occupancy of MRs compared to GRs in the hippocampus (de Kloet et al., 1999; Herbert et al., 2006; Ferguson and Sapolsky, 2007) and thus promote cognitive function.

### Gene Expression

In early adulthood, *Bdnf* levels were downregulated in the dorsal and ventral hippocampus of HR ELS mice compared to HR STD animals (**Figures 7A,E**), likely reflecting a downstream effect of exaggerated glucocorticoid stimulation, as it has repeatedly been shown that the BDNF- and the glucocorticoid-signaling pathways are closely interlinked and show bi-directional cross talk (Jeanneteau and Chao, 2013; Daskalakis et al., 2015). In late adulthood, the difference in *Bdnf* levels between HR ELS and STD mice was reduced, due to a downward shift in the expression levels of HR STD animals (**Figures 7B,F**). Overall, HR mice had lower *Bdnf* levels than LR mice, confirming previous findings at the level of proteins in the hippocampus in HR animals (Knapman et al., 2010b). Being an important mediator of neural growth and survival, as well as of synaptic plasticity (Huang and Reichardt, 2001; Daskalakis et al., 2015), BDNF plays a central role in the underlying processes of learning and memory (Cunha et al., 2010). Thus, a reduced availability of BDNF, as observed in HR mice and in HR ELS in particular, may contribute to the impaired cognitive performance of these animals. ELS is known to impact on BDNF expression in the hippocampus (Liu et al., 2000), reinforcing the association with ELS-induced cognitive deficits that have been described (Daskalakis et al., 2015). In line with this, our data showed a significant positive correlation of BDNF mRNA expression in the dorsal hippocampus and cognitive performance in the object recognition test. The reason this association was not significant in the Y-maze test may be that spatial discrimination in the Y-maze test relies mainly on the hippocampal formation, while the object recognition test performance also depends on the prefrontal and parahippocampal cortex, where BDNF is a key regulator of neuronal function.

The reduced hippocampal expression of BDNF mRNA most likely reflects a dynamic epigenetic signature of ELS and augmented stress hormone signaling, rather than a genetically encoded difference between the mouse lines, since in early adulthood, HR ELS and STD mice showed condition-dependent differences in *Bdnf* expression levels. Glucocorticoids can have profound effects on the regulation of a range of transcription factors, the epigenome, and mircoRNAs, suggesting a wide array of potential programming pathways (de Kloet et al., 2009; McGowan et al., 2009; Suri and Vaidya, 2013).

Changes in the CRH system have also repeatedly been implicated in the adverse effects of ELS (Avishai-Eliner et al., 2001; Rice et al., 2008; Ivy et al., 2010; Korosi et al., 2010; Wang et al., 2013; Fuge et al., 2014; Liao et al., 2014). In early adulthood, there was an upregulation of *Crh* in the dorsal hippocampus of ELS-exposed HR mice compared to HR STD animals (McIlwrick et al., 2016, also shown in **Figure 8A**), which we now observed to be stable into late adulthood (**Figure 8B**). However, at both ages, we detected no evidence for changes in the expression of *Crhr1* in the hippocampus (McIlwrick et al., 2016, and **Figures 8C,D,G,H**).

An increased tone of CRH activity in the hippocampus could constitute a further pathway contributing to deficits in hippocampus-dependent cognitive tasks, as CRH-signaling has been implicated in dendritic remodeling (Chen et al., 2008, 2012) and is a prominent target for ELS-induced epigenetic programming (Murgatroyd and Spengler, 2011; Karsten and Baram, 2013). The significant negative correlation of dorsal hippocampal *Crh* expression and cognitive performance in aged animals provides evidence for this association. As discussed above, task performance in the Y-maze test is highly dependent in intact hippocampal functioning, while the object recognition test also recruits different cortical areas. This explains the stronger impact of increased hippocampal *Crh* expression on the spatial memory in the Y-maze than on recognition memory in the object recognition test. In LR mice, no ELS-associated changes in *Crh* and *Crhr1* expression were observed in the dorsal hippocampus during early adulthood (McIlwrick et al., 2016, also shown in **Figures 8A,C**), but a downregulation of both genes became evident in late adulthood (**Figures 8B,D**), coinciding with somewhat enhanced cognitive performance of LR ELS mice (**Figure 4B**). In conclusion, our data suggests that ELS exposure triggers a range of neuroendocrine and molecular alterations, the effects of which emerge gradually in early adulthood and strongly depend on the animal’s inherited predisposition for high or low stress reactivity.

### Shortcomings and Future Directions

The selection of appropriate behavioral tests is critical to reliably measure small effects of experimental manipulations in behaving animals. To assess cognitive function in early and late adulthood we used the Y-maze test at both time points, but supplemented this with different behavioral tasks (i.e., the WCM was used only in early adulthood; the object recognition test was used only in late adulthood), which creates some asymmetry in the data. The reason why we did not repeat the WCM in the late adulthood animal cohort was that this test presupposes the animal’s ability to navigate using visual cues and albino mice are poorly equipped for vision-based tasks (Brown and Wong, 2007). Hence, animals of the SR mouse model in general performed relatively poorly compared to, e.g., wild type C57Bl6/N mice (Kleinknecht et al., 2012) in the WCM. In addition, the WCM is a relatively stressful test, due to the need for the animals to swim in water, which may impact on the animal’s performance. Therefore, we decided to use a less stressful and less vision-dependent test in the late adulthood cohort of animals. Since the Y-maze test was identical at both time points and the results concurred well with the results of both other cognitive tasks, we believe that our conclusions regarding changes in cognitive function from early to late adulthood are nonetheless valid and justifiable.

In the presented work, we investigated the effects of ELS in male mice, only. However, the clinical reality shows that women are at a twofold increased risk for affective and stress-related disorders (Gater et al., 1998; Castle, 2007). Several factors may play a role in this enhanced vulnerability, including differences in neuroendocrine regulation and interaction of reproductive hormones (Seeman, 1997; Young et al., 2001; Bale, 2006), which may be exacerbated through ELS experiences. As the findings from male animals are not necessarily directly transferable to females and therefore only provide limited information regarding large parts of the patient population, future studies should include female subjects in the investigation.

In recent years, several gene variants that contribute to individual risk or resilience have been identified, including *Nr3c1* (Wust et al., 2009), *Nr3c2* (DeRijk et al., 2006), *Fkbp5* (Ising et al., 2008), *Crhr1* (Clarke and Schumann, 2009), *Crhbp* (Wang et al., 2007), *Gabra6* (Uhart et al., 2004), and *Slc6a4* (Way and Taylor, 2010). It would contribute to our understanding of the gene × environment interaction described here to have a better knowledge about the genomic sequence of the three SR mouse lines, in order to seek confirmation for some of the known risk polymorphisms and to detect new potential candidates. Moreover, an analysis of the methylation status of candidate genes may add valuable information about epigenetic changes induced by ELS in the three SR mouse lines.

## Conclusion

Taken together, we present evidence showing that the lasting effects of ELS on cognitive function can differ greatly between individuals and that one key determinant of the long-term outcome is the individual’s inherited predisposition for high or low stress reactivity. The SR mouse model provided us with an ideal tool to investigate the role of innate differences in neuroendocrine HPA axis function in this gene × environment interaction. Using this animal model, we were able to show that, while HR mice display cognitive deficits emerging in early adulthood, accompanied by a hyper-reactive HPA axis and lasting changes in the regulation of *Crh* and *Bdnf* transcripts, LR mice appear to be largely protected against these adverse effects of ELS. Epigenetic processes programming the reactivity of the HPA axis are likely to be involved in shaping these divergent outcomes. Thus, our findings contribute to advancing our understanding of factors influencing the vulnerability or resilience to early-life adversity, and hence to stress-related psychopathologies. Future studies using the SR mouse model could yield valuable insights into the molecular mechanisms underlying resilience and vulnerability, including the genetic and epigenetic underpinnings. An improved understanding of how ELS interacts with an inherited predisposition to program individuals for increased stress sensitivity and risk for affective disorders could guide the design of future treatment options by reversing or otherwise targeting these pathological processes.

## Author Contributions

All listed authors contributed to this manuscript: SM, TP, AC, and CT. The study was designed by SM and CT. SM performed the experiments and prepared the manuscript. TP assisted with behavioral testing in the late adulthood animal cohort. CT edited the manuscript and supervised the study. AC gave advice regarding the format of the manuscript and data presentation. All authors approved the final version of the manuscript.

## Conflict of Interest Statement

The authors declare that the research was conducted in the absence of any commercial or financial relationships that could be construed as a potential conflict of interest.

## Supplementary Material

FIGURE S1 **Experimental timeline.** The timeline provides an overview of the assessments which were taken at the different time points throughout the study. To cover the range of different tests, overall, animals from three experimental cohorts (I, II, and III) were employed in this study. A small subset of the data has been published previously (McIlwrick et al., 2016) and is indicated here by ^∗^. Data that is a new replication of earlier findings is indicated here by ^∗∗^.
