# Supplementary figures and images for: Late-Onset Cognitive Impairments after Early-Life Stress Are Shaped by Inherited Differences in Stress Reactivity
Source: Front Cell Neurosci. 2017 Feb 14;11:9. doi: 10.3389/fncel.2017.00009 (PMC5306385; doi:10.3389/fncel.2017.00009)

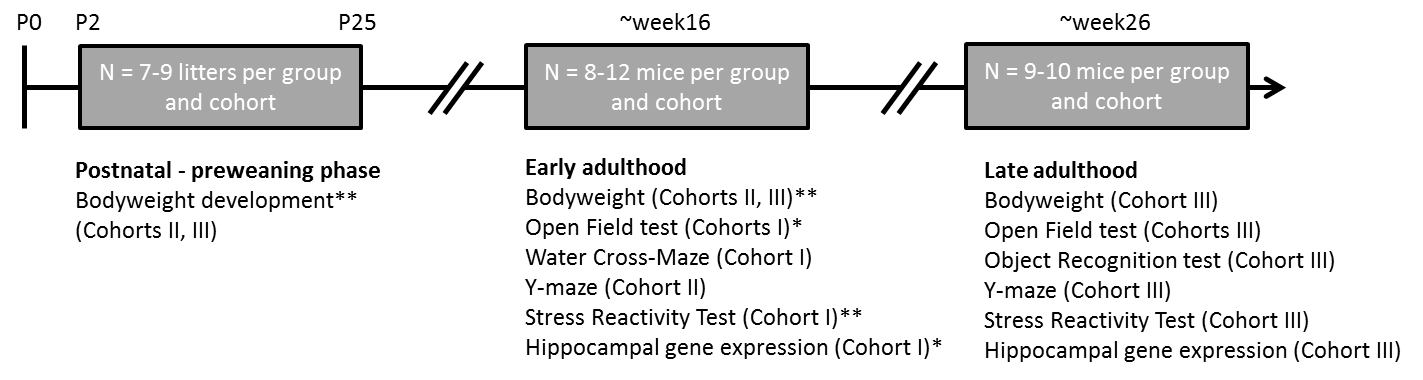

Supplement: FIGURE S1 — Experimental timeline. The timeline provides an overview of the assessments which were taken at the different time points throughout the study. To cover the range of different tests, overall, animals from three experimental cohorts (I, II, and III) were employed in this study. A small subset of the data has been published previously (McIlwrick et al., 2016) and is indicated here by ∗. Data that is a new replication of earlier findings is indicated here by ∗∗. [file Image_1.PNG]
